# Broad-Spectrum Inhibitors for Conserved Unique Phosphoethanolamine Methyltransferases in Parasitic Nematodes Possess Anthelmintic Efficacy

**DOI:** 10.1128/aac.00008-23

**Published:** 2023-05-22

**Authors:** Xuejin Zhang, Leonor Sicalo Gianechini, Kun Li, Ray M. Kaplan, William H. Witola

**Affiliations:** a Department of Pathobiology, College of Veterinary Medicine, University of Illinois Urbana-Champaign, Urbana, Illinois, USA; b Department of Infectious Diseases, University of Georgia, Athens, Georgia, USA; c Institute of Traditional Chinese Veterinary Medicine, MOE Joint International Research Laboratory of Animal Health and Food Safety, College of Veterinary Medicine, Nanjing Agricultural University, Nanjing, China; d Pathobiology Department, School of Veterinary Medicine, St. George’s University, Grenada, West Indies

**Keywords:** helminths, phosphatidylcholine biosynthesis, conserved enzymes, molecular targets, novel anthelmintics

## Abstract

In humans, nematode infections are prevalent in developing countries, causing long-term ill health, particularly in children. Worldwide, nematode infections are prevalent in livestock and pets, affecting productivity and health. Anthelmintic drugs are the primary means of controlling nematodes, but there is now high prevalence of anthelmintic resistance, requiring urgent identification of new molecular targets for anthelmintics with novel mechanisms of action. Here, we identified orthologous genes for phosphoethanolamine methyltransferases (PMTs) in nematodes within the families *Trichostrongylidae*, *Dictyocaulidae*, *Chabertiidae*, *Ancylostomatoidea*, and *Ascarididae*. We characterized these putative PMTs and found that they possess *bona fide* PMT catalytic activities. By complementing a mutant yeast strain lacking the ability to synthesize phosphatidylcholine, the PMTs were validated to catalyze the biosynthesis of phosphatidylcholine. Using an *in vitro* phosphoethanolamine methyltransferase assay with PMTs as enzymes, we identified compounds with cross-inhibitory effects against the PMTs. Corroboratively, treatment of PMT-complemented yeast with the PMT inhibitors blocked growth of the yeast, underscoring the essential role of the PMTs in phosphatidylcholine synthesis. Fifteen of the inhibitors with the highest activity against complemented yeast were tested against Haemonchus contortus using larval development and motility assays. Among them, four were found to possess potent anthelmintic activity against both multiple drug-resistant and susceptible isolates of *H. contortus*, with IC_50_ values (95% confidence interval) of 4.30 μM (2.15–8.28), 4.46 μM (3.22–6.16), 28.7 μM (17.3–49.5), and 0.65 μM (0.21–1.88). Taken together, we have validated a molecular target conserved in a broad range of nematodes and identified its inhibitors that possess potent *in vitro* anthelmintic activity.

## INTRODUCTION

Parasitic nematode infections are common among humans, with over 300 species that are known to infect humans (1, 2). It is estimated that around 3.5 billion humans are infected by the most prevalent nematodes, including Ascaris lumbricoides, Ancylostoma duodenale, Necator americanus and Trichuris trichiura (3, 4). In humans, nematode infections are highly prevalent in developing countries and are associated with low-income groups leading to health problems and thus require sustained control efforts (5). On the other hand, in animal health, nematode infections are highly prevalent worldwide and are among the most economically important factors affecting production in grazing livestock, costing the global livestock industry billions of dollars annually in lost production and treatment expenses (6–8).

The main mode of control of parasitic nematode infections in humans and animals is through the administration of anthelmintic drugs. At present, only a few main classes of anthelmintic drugs are commercially available, including benzimidazoles, imidazothiazoles, tetrahydropyrimidines, macrocyclic lactones, amino-acetonitrile derivatives (AAD), salicylanilides, and cyclooctadepsipeptides (9–12). However, anthelmintic resistance in livestock nematodes has become a serious global problem (13). Although less prevalent than in livestock, anthelmintic treatment failures have also been reported in humans for most commonly used anthelmintics (14–17). With the increasing prevalence of anthelmintic resistance, there is an urgent need for concerted efforts to identify novel strategies for developing new effective drugs (18).

Within the infected host, nematodes are prolific egg layers, requiring active biogenesis of nematode plasma membranes in which phospholipids, particularly phosphatidylcholine, are major constituents (19, 20). Phosphatidylcholine is important for maintaining the plasma membrane architecture and functions, as well as for cellular signal transduction, thus indicating that it is indispensable for survival of parasitic nematodes (21). Significant disparities in fundamental biochemical and metabolic pathways of phospholipids between parasitic nematodes and their animal hosts exist. A plant-like phosphobase methylation pathway involving the three-step *S*-adenosyl-l-methionine (SAM)-dependent methylation of phosphoethanolamine to phosphocholine for the biosynthesis of phosphatidylcholine has been characterized and found to be essential for the protozoan parasite, Plasmodium falciparum (22, 23), as well as for the free-living nematode Caenorhabditis elegans (24) and the livestock parasitic nematode Haemonchus contortus (25–27).

The phosphobase methylation step in C. elegans and *H. contortus* is catalyzed by two phosphoethanolamine methyltransferases, PMT1 and PMT2, that sequentially methylate phosphoethanolamine (PE) to phosphocholine (24, 25, 28). PMT1 possesses an N-terminal methyltransferase domain that catalyzes the first methylation of PE to phosphomonomethylethanolamine (PMME), while PMT2 possesses the C-terminal domain that catalyzes the subsequent methylation steps of converting PMME to phosphodimethylethanolamine (PDME) and then methylating PDME to phosphocholine, which enters the Kennedy pathway to be converted to phosphatidylcholine (28, 29). Because this phosphobase methylation step does not exist in mammalian cells, it is considered an attractive nematode-specific molecular target for the development of a novel class of efficacious anthelmintic drugs (26, 27, 30–32).

Here, we used genetic and biochemical approaches to functionally characterize putative phosphoethanolamine methyltransferases (PMTs) from various families of parasitic nematodes and identified their broad-spectrum inhibitors that we then tested for *in vitro* anthelmintic efficacy.

## RESULTS

### Orthologous putative PMTs in parasitic nematodes of different families.

**FIG 1:**
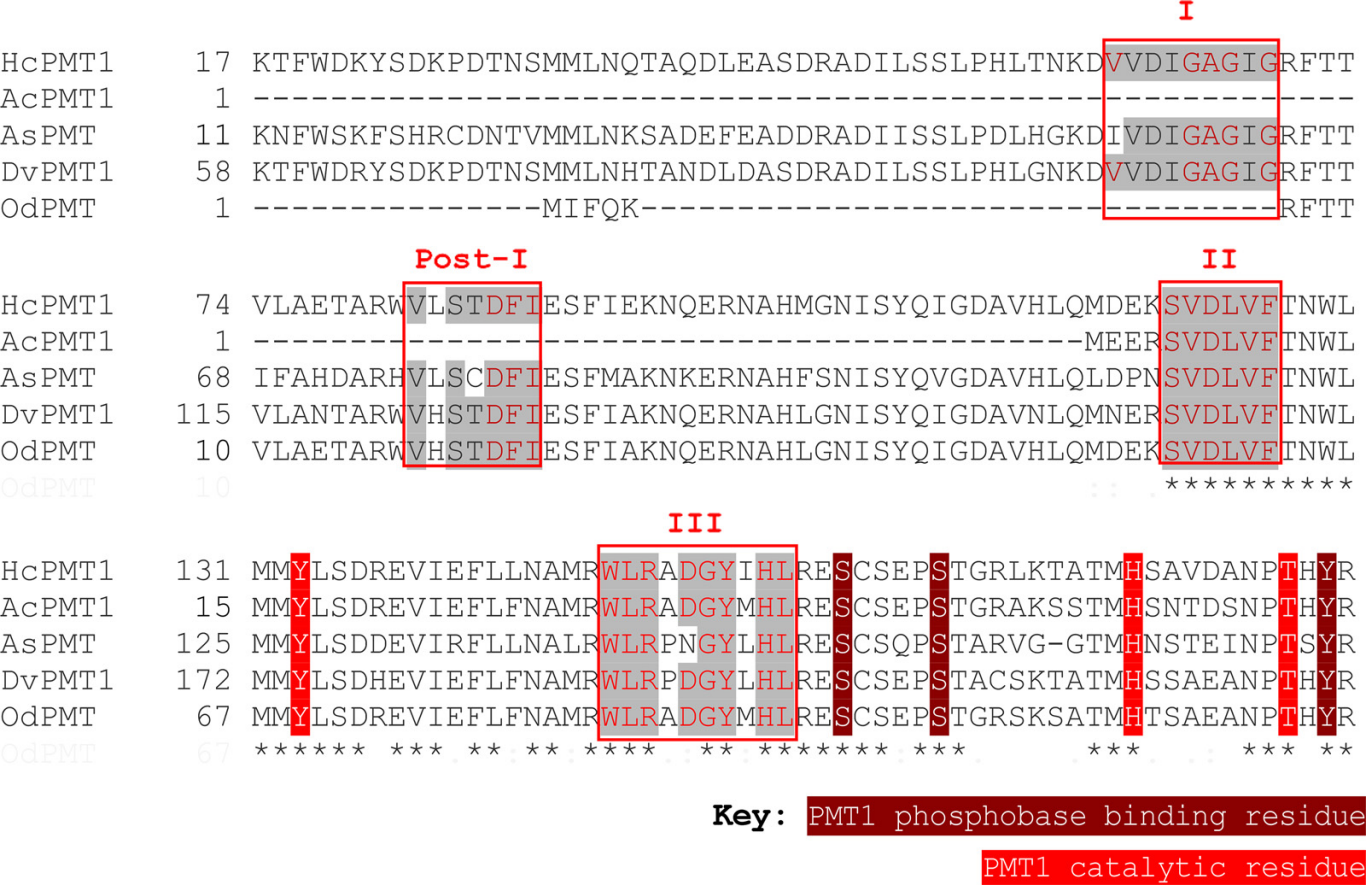
Multiple sequence alignment of Haemonchus contortus PMT1 (HcPMT1, 4KRG_A), Ancylostoma duodenale PMT1 (AcPMT1, KIH60772.1), Ascaris suum PMT1 (AsPMT1, ERG79882.1), Dictyocaulus viviparus PMT1 (DvPMT1, KJH50371.1), and Oesophagostomum dentatum PMT1 (OdPMT1, KHJ94304.1) by using TCoffee (http://tcoffee.crg.cat/apps/tcoffee/do:regular). Four *S*-adenosyl methionine (SAM)-binding motifs that define the methyltransferases domains are labeled I, Post-I, II, and III and are boxed as previously described (21, 29). Asterisks (*) indicate positions with a single, fully conserved residue.

**FIG 2:**
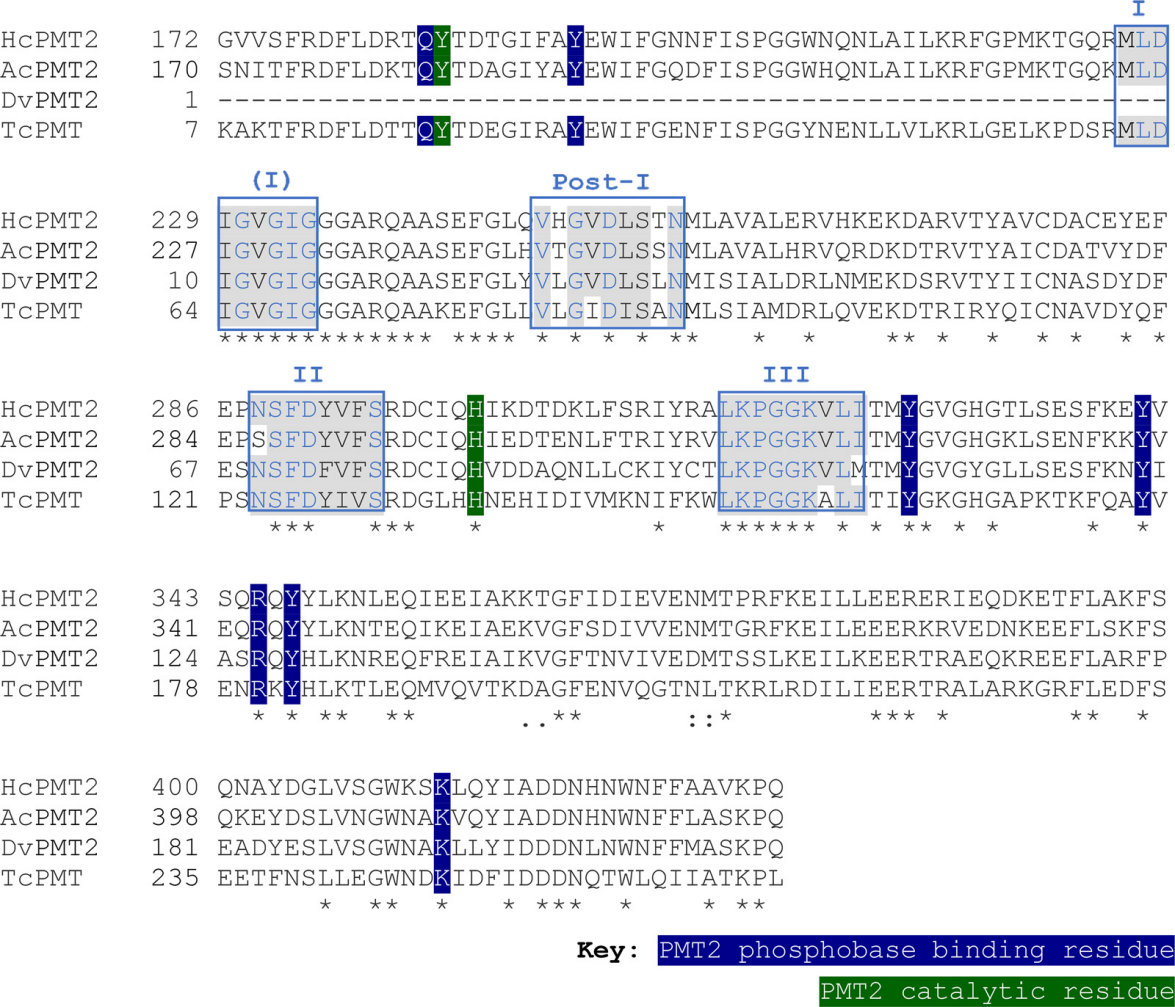
Multiple sequence alignment of *Haemonchus contortus* PMT2 (HcPMT2, 4KRI_A), Ancylostoma ceylanicum PMT2 (AcPMT2, EPB71549.1), *Dictyocaulus viviparous* PMT2 (DvPMT2, KJH40940.1), and Toxocara *canis* PMT2 (TcPMT2, KHN87001.1) by using Tcoffee (http://tcoffee.crg.cat/apps/tcoffee/do:regular). Four *S*-adenosyl methionine (SAM)-binding motifs that define the methyltransferases domains are labeled I, Post-I, II, and III and boxed as previously described (21, 29). Asterisks (*) indicate positions with a single, fully conserved residue.

Despite the limited genome databases for parasitic nematodes, we performed BLAST searches and identified orthologues of *H. contortus* PMT 1 and 2 (HcPMT1 and HcPMT2) in other parasitic nematodes within the families *Trichostrongylidae*, *Dictyocaulidae*, *Chabertidae*, *Ancylostomatoidea*, and *Ascarididae*. *H. contortus* HcPMT1 possesses N-terminal methyltransferase domains that catalyze the first step of methylating phosphoethanolamine (PE) to phosphomonomethylethanolamine (PMME) (29). We identified gene orthologues containing these domains in nematode genera, including *Oesophagostomum*, *Ancylostoma*, *Dictyocaulus*, and *Ascaris* (Fig. 1). In some of these same genera and others, we also identified genes that are orthologous to HcPMT2 (Fig. 2). HcPMT2 possesses C-terminal domains that function to catalyze the subsequent methylation steps of converting PMME to PDME and methylating PDME to phosphocholine (28, 29). A coding gene sequence (orthologous to HcPMT1) from *Ancylostoma duodenale* is 1,026 bp long and encodes a 341-amino-acid polypeptide (GenBank accession number KIH60772.1) with an estimated molecular weight of 37.6 kDa and is here named AcPMT1 (Fig. 1). In addition, from Ancylostoma ceylanicum, we found a 1,296-bp coding gene sequence that is orthologous to HcPMT2 that encodes a 431-amino-acid polypeptide (GenBank accession number EPB71549.1) with an estimated molecular weight of 47.5 kDa and is here called AcPMT2 (Fig. 2). The lungworm (Dictyocaulus viviparus) genome contained coding sequences of both HcPMT1 and HcPMT2 orthologues, namely, DvPMT1 (1,452 bp) and DvPMT2 (645 bp). DvPMT1 and DvPMT2 code for 483- and 214-amino-acid polypeptides (GenBank accession numbers KJH50371.1 and KJH40940.1), with calculated molecular weights of 53.3 and 23.7 kDa, respectively (Fig. 1 and 2).

The available genomes of three nematodes, namely, *Ascaris suum*, *Oesophagostomum dentatum*, and Toxocara *canis*, each yielded a single PMT orthologue. The *A. suum* genome contained a coding sequence (orthologous to HcPMT1) that is 1,383 bp long, encodes a 460-amino-acid polypeptide (WormBase Parasite ID: LK871972.1) with a calculated molecular weight of 47.5 kDa, and is here named AsPMT (Fig. 1). The genome of *O. dentatum* possessed a coding sequence which is 1,119 bp long and encodes a 372-amino-acid polypeptide orthologous to HcPMT1 (GenBank accession number KHJ94304.1) with a calculated molecular weight of 41 kDa and is here called OdPMT (Fig. 1). On the other hand, the *T. canis* genome contained a HcPMT2 orthologue with a 807-bp coding sequence for a 268-amino-acid polypeptide (GenBank accession number KHN87001.1) with a calculated molecular weight of 29.6 kDa and is here named TcPMT (Fig. 2).

The amino acid sequences of putative AcPMT1, AsPMT1, DvPMT1, and OdPMT share 77.13, 53.59, 76.87, and 77.42% identity with HcPMT1, respectively, and <20% identity with HcPMT2. On the other hand, the putative AcPMT2, DvPMT2, and TcPMT share about 66.82, 66.82, and 51.87% identity with HcPMT2, respectively, but only 20% identity with HcPMT1. The putative AcPMT1, DvPMT1, AsPMT, and OdPMT were found to possess the conserved methyltransferase catalytic domains as well as residues that are important for substrate (phosphoethanolamine) and cosubstrate (SAM) binding (Fig. 1). On the other hand, the putative AcPMT2, DvPMT2, and TcPMT possessed catalytic domains with the substrate and cosubstrate binding residues (Fig. 2) that have been reported to be conserved in HcPMT2 (29).

### Nematode-specific PMTs catalyze SAM-dependent methylation of phosphoethanolamine and depict Michaelis-Menten kinetics.

The recombinant PMTs were expressed in Escherichia coli as N-terminal His_6_-tagged proteins and purified using nickel affinity column chromatography. Analysis of the purified proteins by SDS-PAGE depicted the molecular weights of AcPMT1, AcPMT2, AsPMT, DvPMT1, DvPMT2, OdPMT, and TcPMT to be approximately 38, 48, 51, 53, 24, 41, and 30 kDa, respectively, consistent with their predicated molecular weights (see Fig. S1 in the supplemental material). To analyze the *in vitro* SAM-dependent methyltransferase activities of the recombinant proteins, we used a fluorescence-based assay with PE as the substrate, SAM as the methyl donor, and individual purified recombinant nematode PMT1 proteins as enzymes. The concentrations of PE and SAM were fixed at 200 and 100 μM, respectively, while the catalytic activity of each protein was measured at various concentrations. All the putative PMT1 proteins that were orthologous to HcPMT1 (AcPMT1, AsPMT, DvPMT1, and OdPMT) depicted concentration-dependent increase in catalytic activity (see Fig. S2), consistent with our previous observations using HcPMT1 as enzyme in the assay (27). Because the genome database of *T. canis* only yielded a single PMT (TcPMT) that was orthologous to HcPMT2 instead of HcPMT1, we tested this protein for catalytic activity of methylating PE. Interestingly, we found that unlike previous observations with HcPMT2 in which no significant catalytic activity was observed using PE as the substrate (27), TcPMT showed concentration-dependent methyltransferase catalytic activity of methylating PE. The derived optimal concentrations of AcPMT1, AsPMT, DvPMT1, OdPMT, and TcPMT were 28.5, 22.5, 99, 28.5, and 50 ng/μL, respectively.

Subsequently, steady-state kinetic parameters of the putative PMTs on SAM and PE were determined by varying the SAM concentration, while maintaining a fixed concentration of PE and vice versa. The catalytic activities of the recombinant proteins (AcPMT1, AsPMT, DvPMT1, OdPMT, and TcPMT) were consistent with Michaelis-Menten kinetics on the substrate, PE (Table 1), and the cosubstrate, SAM (Table 2).

**TABLE 1:** Comparison of different PMT proteins’ enzymatic kinetic parameters on phosphoethanolamine

Enzyme	Mean ± SEM			*k*_cat_/*K_m_* (s^−1^ M^−1^)
	*K_m_* (μM)	*V*_max_ (nmol^−1^ mg^−1^ min^−1^)	*k*_cat_ (min^−1^)	
HcPMT1	73.37 ± 54.91	629.10 ± 160.90	1,066.30 ± 72.70	2.42 × 10^5^
AcPMT1	1,079.00 ± 65.11	4,738.00 ± 764.50	7,071.00 ± 1,141.00	1.09 × 10^5^
AsPMT	99.93 ± 36.63	184.60 ± 27.93	194.30 ± 29.40	3.20 × 10^4^
DvPMT1	308.00 ± 41.68	2,378.00 ± 237.60	966.80 ± 96.59	5.23 × 10^4^
OdPMT	80.97 ± 12.83	231.10 ± 14.92	335.00 ± 21.62	6.90 × 10^4^
TcPMT	85.00 ± 77.76	1,031.00 ± 366.30	624.70 ± 221.90	1.22 × 10^5^

**TABLE 2:** Comparison of different PMT proteins’ enzymatic kinetic parameters on *S*-adenosyl-l-methionine

Enzyme	Mean ± SEM			*k*_cat_/*K_m_* (s^−1^ M^−1^)
	*K_m_* (μM)	*V*_max_ (nmol^−1^ mg^−1^ min^−1^)	*k*_cat_ (min^−1^)	
HcPMT1	18.34 ± 3.80	2,544.00 ± 122.20	4,312.00 ± 203.70	3.91 × 10^6^
AcPMT1	27.33 ± 3.58	2,172.00 ± 86.63	3,241.00 ± 120.90	1.98 × 10^6^
AsPMT	13.66 ± 0.62	3,207.00 ± 34.95	8,668.00 ± 94.47	1.06 × 10^7^
DvPMT1	35.02 ± 5.05	444.60 ± 50.05	237.70 ± 12.87	1.13 × 10^5^
OdPMT	48.95 ± 7.07	3,131.00 ± 204.30	2,723.00 ± 177.70	9.27 × 10^5^
TcPMT	20.31 ± 2.23	1,083.00 ± 32.36	656.50 ± 19.61	5.39 × 10^5^

### Expression of nematode PMTs in *pem1Δpem2Δ Saccharomyces cerevisiae* mutant yeast cells.

**FIG 3:**
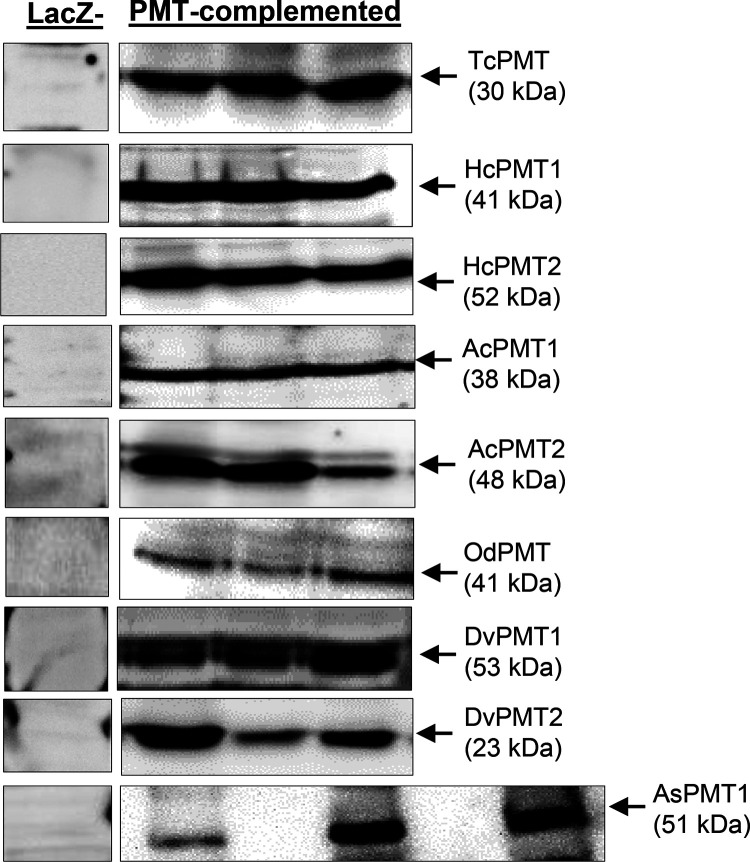
Western blotting of the expression of nematode PMT proteins in complemented yeast. Lysates of cultured complemented yeast cells induced to express PMT transgenes were fractionated by SDS-PAGE, followed by immunoblotting using affinity-purified antisera against respective nematode PMTs. For each complemented yeast strain, lysates generated from three individual yeast colonies were analyzed, and the expressed PMT protein band is shown in three separate lanes (PMT-complemented). The control lane was loaded with yeast lysate from LacZ-complemented yeast (LacZ-). The expected molecular weight is indicated in kDa in parenthesis below the name of the PMT. TcPMT, Toxocara *canis* PMT; HcPMT, *Haemonchus contortus* PMT; AcPMT, Ancylostoma ceylanicum PMT; OdPMT, *Oesophagostomum dentatum* PMT; DvPMT, Dictyocaulus viviparus PMT; AsPMT, *Ascaris suum* PMT.

Yeast cells inherently lack PMT activity but express phosphatidylethanolamine *N*-methyltransferase (PEMT1 and PEMT2) proteins that are required for the three-step methylation of phosphatidylethanolamine (PtdEtn) to phosphatidylcholine (PtdCho). Deletion of both PMETs is lethal for yeast unless exogenous choline is provided in growth medium (33, 34). Therefore, to determine the *bona fide* role of nematode PMTs, we analyzed their ability to complement the loss of PtdCho biosynthesis in *pem1Δpem2Δ* S. cerevisiae mutant strain (35). The coding sequences of nematode PMTs (HcPMT1, HcPMT2, AcPMT1, AcPMT2, AsPMT, DvPMT1, DvPMT2, OdPMT, and TcPMT) and that of LacZ (as a negative control) were transformed and expressed in *pem1*Δ/*pem2*Δ S. cerevisiae under the control of the GAL1-inducible promoter. Western blot analysis using affinity-purified polyclonal antibodies raised in rats against corresponding nematode PMT recombinant proteins showed that the antisera recognized their respective protein bands (with predicted molecular weights) in whole-cell lysates of PMT-complemented *pem1*Δ/*pem2*Δ S. cerevisiae grown under induction conditions (minimal medium with 2% galactose supplemented with 1 mM ethanolamine) (Fig. 3). In yeast transformed with two PMTs (HcPMT1+HcPMT2, AcPMT1+AcPMT2, or DvPMT1+DvPMT2), the expression of both proteins in the same yeast was detected (Fig. 3). No PMT-specific bands were detected in the lysate of LacZ-complemented yeast (Fig. 3).

### Complementation of *pem1*Δ/*pem2*Δ *S. cerevisiae* with nematode PMTs rescues growth in the absence of choline.

**FIG 4:**
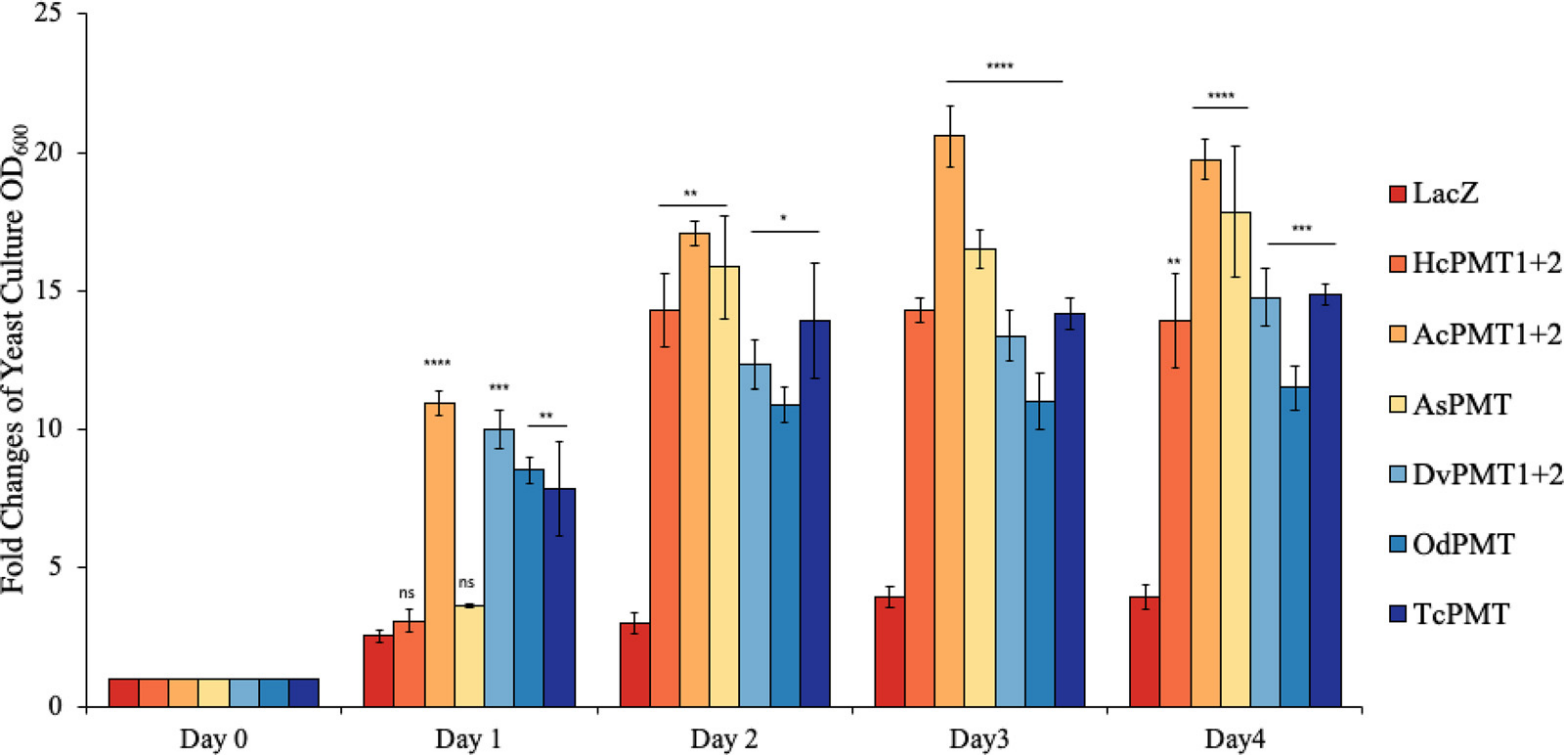
Analysis of the growth of nematode PMT-complemented *pem1Δpem2Δ* S. cerevisiae in culture over time. The growth of *pem1Δpem2Δ* S. cerevisiae expressing the respective nematode PMTs or LacZ growth medium supplemented with ethanolamine and galactose was determined over a 4-day culture period. TcPMT, yeast expressing Toxocara *canis* PMT; HcPMT1+2, yeast expressing Haemonchus contortus PMT1 and PMT2; AcPMT1+2, yeast expressing Ancylostoma ceylanicum PMT1 and PMT2; OdPMT, yeast expressing Oesophagostomum dentatum PMT; DvPMT1+2, yeast expressing Dictyocaulus viviparus PMT1 and PMT2; AsPMT, yeast expressing Ascaris suum PM; LacZ, yeast expressing *lacZ* gene (control). The data shown represent means from three independent experiments with standard error bars.

The choline auxotroph *pem1*Δ/*pem2*Δ S. cerevisiae is unable to grow in medium lacking exogenous choline. Therefore, we cultured the nematode PMT-complemented *pem1*Δ/*pem2*Δ S. cerevisiae mutant yeast in medium lacking choline but supplemented with 1 mM exogenous ethanolamine in order to determine whether nematode PMT-catalyzed methylation of PE to phosphocholine would lead to *de novo* biosynthesis of PtdCho and rescue yeast growth. By measuring the growth of the yeast cells over 3 days, we found that the LacZ-complemented *pem1*Δ/*pem2*Δ S. cerevisiae strain did not show significant growth over time (Fig. 4), consistent with previous findings that lack of both PEM1 and PEM2 is lethal for S. cerevisiae unless choline is provided in medium (33, 34). Intriguingly, we found that all the PMT-complemented *pem1*Δ/*pem2*Δ S. cerevisiae, depicted significant progressive growth overtime (Fig. 4). Notably, there were some variations in the magnitude of growth conferred by different nematode PMTs, with AcPMT1+AcPMT2 complementation depicting the highest growth, followed by AsPMT, TcPMT, HcPMT1+HcPMT2, DvPMT1+DvPMT2, and TcPMT, in that order (Fig. 4). Variations in magnitude of growth can be attributed to the variations in levels of expression of the respective PMTs and the corresponding amount of PtdCho synthesized in the yeast.

**FIG 5:**
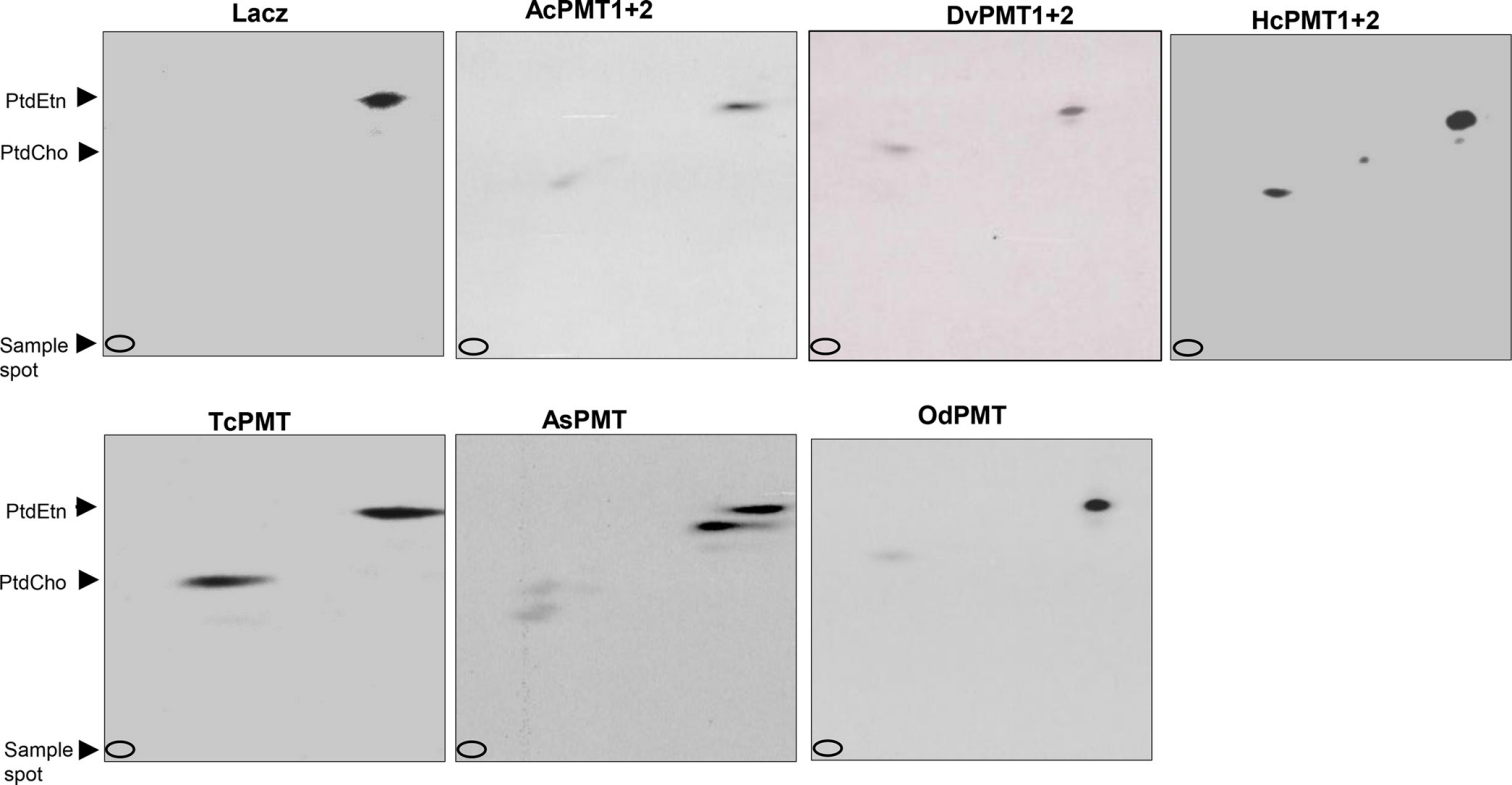
2D-TLC analysis of the synthesis of phosphatidylcholine and phosphatidylethanolamine using [^14^C]ethanolamine as the substrate in *pem1Δ/pem2Δ* yeast complemented with nematode PMT(s). The complemented yeast strains were grown in medium with [^14^C]ethanolamine hydrochloride and galactose for 3 days, and lipids were extracted by the Folch method. The organic phase of the lipid extracts was resolved by 2D-TLC, and signals were generated by autoradiography. PtdEtn and PtdCho, positions of phosphatidylethanolamine and phosphatidylcholine, respectively; LacZ, yeast expressing LacZ gene (control); AcPMT1+2, yeast expressing Ancylostoma ceylanicum PMT1 and PMT2; DvPMT1+2, yeast expressing Dictyocaulus viviparus PMT1 and PMT2; HcPMT1+2: yeast expressing *Haemonchus contortus* PMT1 and PMT2; TcPMT, yeast expressing Toxocara canis PMT; AsPMT, yeast expressing *Ascaris suum* PMT; OdPMT, yeast expressing *Oesophagostomum dentatum* PMT. The data shown are representative of three independent experiments.

To confirm that nematode PMT-complemented *pem1*Δ/*pem2*Δ S. cerevisiae was able to synthesize PtdCho, we performed radioisotope incorporation assays by supplementing the yeast with [^14^C]ethanolamine hydrochloride, along with cold ethanolamine, in medium lacking choline. Two-dimensional thin-layer chromatography (2D-TLC) analysis of the organic-phase lipid extracts from the yeast showed the presence of both PtdEtn and PtdCho with incorporated radioisotope in the PMT-complemented *pem1*Δ/*pem2*Δ S. cerevisiae strains (Fig. 5). To allow growth of the LacZ-complemented yeast, in addition to hot and cold ethanolamine, the medium was also supplemented with choline. As expected, unlike the PMT-completed yeast, LacZ-complemented yeast had radioisotope incorporated only in PtdEtn, synthesized through the CDP-ethanolamine pathway (Fig. 5). The positions and patterns of the detected bands of PtdEtn and PtdCho were consistent with previous observations in studies using *Plasmodium* PMT (22). These results indicate that the yeast mutant was able to use ethanolamine to synthesize PtdEtn which cannot be converted to PtdCho because of the lack of PEMT1 and PEMT2 enzyme. However, the expression of the exogenous nematode PMTs catalyzed the methylation of ethanolamine to phosphocholine, which was then converted to PtdCho via the Kennedy Pathway, as evidenced by the presence of a band of radiolabeled PtdCho in the complemented mutant yeast (Fig. 5). Noteworthy, the intensity of the PtdCho band varied among the different PMT-complemented strains, which can be attributed to the differences in the expression levels of the nematode PMTs and their activity in the yeast cells. Together, these results validate the role of nematode PMTs in the biosynthesis of PtdCho.

### Natural compounds with broad-spectrum inhibitory activity against nematode PMTs.

To identify chemical compounds that have inhibitory effect against the methyltransferase activity of nematodes PMTs, we screened a Natural Products Set IV library consisting of 419 compounds (see Table S1) from the National Cancer Institute Open Chemical Repository. Because HcPMT1 had earlier been characterized (25, 27, 29), we used it as an enzyme in the methyltransferase assay for the initial screening of the compound library. Of 419 compounds screened, we identified 45 that showed a ≥50% inhibitory effect (*P < *0.05) against the catalytic activity of recombinant HcPMT1 at 40 μM (see Fig. S3 and S4). The rest of the compounds either had <50% inhibitory effect or augmented the catalytic activity of recombinant HcPMT1 and were thus not pursued further. Prior to evaluation of their inhibitory effect on the rest of the nematode PMTs, the identified HcPMT1 inhibitors were tested for mammalian cell cytotoxicity at and above the concentrations that inhibited HcPMT1. Among the 45 HcPMT1 inhibitors, 20 compounds showed no cytotoxicity in HCT-8 cells (American Type Culture Collection CCL244) at the highest concentration (160 μM) tested. Those compounds included NSC12097, NSC87511, NSC133100, NSC56410, NSC177858, NSC333856, NSC620709, NSC35676, NSC24872, NSC99791, NSC156219, NSC186301, NSC145612, NSC177383, NSC335989, NSC113497, NSC227186, NSC328426, and NSC320301. The rest of the compounds displayed significant cytotoxicity against HCT-8 cells at ≤40 μM and were thus not pursued further. The nontoxic 20 HcPMT1 inhibitors were next tested for cross-inhibitory activity against other nematode PMTs from *A. duodenale* (AcPMT1), *A. suum* (AsPMT), *D. viviparus* (DvPMT1), *O. dentatum* (OdPMT1), and *T. canis* (TcPMT). For initial screening, all compounds were tested at 40 μM, and seven compounds (NSC87511, NSC133100, NSC56410, NSC62709, NSC35676, NSC145612, and NSC177383) showed significant cross-inhibitory activity against all the five PMTs (see Fig. S5). Subsequently, for each PMT protein, various concentrations of each of the seven compounds were tested, and the enzyme half-maximal inhibitory concentrations (IC_50_^Enzyme^) against the various PMTs’ catalytic activities were derived (Table 3). Alongside that, the seven compounds were also tested for mammalian cell cytotoxicity at various concentrations and their cytotoxicity half-maximal inhibitory concentrations (IC_50_^Cytotoxicity^) derived (Table 3). From these data, the selectivity indexes (SI) for the compounds were calculated (Table 4), and compounds NSC35676, NSC62709, NSC145612, NSC177383, and NSC133100 displayed SI values that were >1 based on the IC_50_^Enzyme^ values of the PMT proteins tested (Table 4), indicating that they were effective PMT cross-inhibitors at nontoxic concentrations. Compounds NSC56410 and NSC87511 had SI values of >1 for four and two of the six PMTs tested, respectively (Table 4).

**TABLE 3:** PMT inhibition IC_50_^Enyme^ and mammalian cell cytotoxicity IC_50_^Cytotoxicity^ values for tested compounds

NSC	Mean IC_50_ (μM) ± SEM						
	Cytotoxicity	Enzyme					
		HcPMT1	AcPMT1	AsPMT	DvPMT1	OdPMT	TcPMT
87511	171.80 ± 8.68	156.70 ± 26.02	65.80 ± 9.78	177.20 ± 15.62	503.50 ± 188.20	487.80 ± 161.70	496.50 ± 184.00
133100	101.80 ± 16.58	27.81 ± 1.71	31.49 ± 2.25	34.05 ± 2.85	88.17 ± 4.14	94.73 ± 5.03	99.10 ± 9.00
56410	188.40 ± 4.19	53.49 ± 5.85	68.41 ± 5.39	58.12 ± 12.95	1,916.00 ± 112.00	210.50 ± 73.54	84.48 ± 6.53
62709	298.90 ± 6.27	27.53 ± 1.97	13.40 ± 1.30	44.31 ± 1.35	29.20 ± 2.13	31.82 ± 1.80	27.59 ± 1.26
35676	566.20 ± 55.94	20.25 ± 1.64	11.55 ± 0.94	12.42 ± 1.10	42.12 ± 3.16	16.75 ± 0.41	45.86 ± 2.90
145612	302.40 ± 9.33	27.91 ± 1.57	27.37 ± 2.92	45.33 ± 1.71	55.12 ± 4.46	33.20 ± 2.26	109.00 ± 12.58
177383	85.47 ± 2.77	21.74 ± 2.05	14.84 ± 2.06	24.60 ± 1.79	47.57 ± 7.41	26.71 ± 2.00	41.27 ± 2.80

**TABLE 4:** Selectivity index values for PMT inhibitors

Compound NSC	SI^*a*^					
	HcPMT1	AcPMT1	AsPMT	DvPMT1	OdPMT	TcPMT
87511	1.10	2.61	0.97	0.34	0.35	0.35
133100	3.67	3.23	3.00	1.15	1.07	1.03
56410	3.52	2.75	3.24	0.10	0.90	2.23
62709	10.86	22.31	6.75	10.24	9.39	10.83
35676	27.96	49.02	45.59	13.44	33.80	12.35
145612	10.83	11.05	6.67	5.49	9.11	2.77
177383	3.93	5.76	3.47	1.80	3.20	2.07

a The selectivity index (SI) was calculated as IC_50_^Cytotoxicity^/IC_50_^Enzyme^.

### Broad-spectrum PMT inhibitors depict concentration-dependent growth-inhibitory effect on PMT-complemented *pem1*Δ/*pem2*Δ *S. cerevisiae*.

To determine the *de novo* efficacy of nematode PMT inhibitors, we performed growth kinetic studies by treating LacZ (control)- and nematode PMT-complemented yeast with various concentrations of the broad-spectrum PMTs inhibitors. We found that compounds NSC56410, NSC62709, NSC177858, NSC133100, NSC35676, NSC87511, and NSC145612 (seven compounds that possess *in vitro* cross-inhibitory activity against nematode PMTs’ catalytic activity) depicted concentration-dependent inhibitory effect on the growth of PMT-complemented mutant yeast (Table 5), while having no or negligible inhibitory effect against LacZ-expressing mutant yeast. The PMT-complemented yeast cells were grown in medium lacking choline but supplemented with ethanolamine which facilitated synthesis of phosphatidylcholine using ethanolamine as precursor via the catalytic activity of nematode PMTs. On the other hand, the LacZ-complemented yeast was grown in medium supplemented with choline, which necessitated the synthesis of phosphatidylcholine using choline as precursor. Therefore, by inhibiting the growth of nematode PMTs-complemented yeast only, it indicates that these compounds were shutting down the biosynthesis of phosphatidylcholine (which is essential for yeast growth) via inhibition of the enzymatic activity of the nematode PMTs. Importantly, all the seven compounds had no effect against the LacZ-complemented yeast when used at concentrations that were effective against PMT-complemented yeast, indicating that they were nontoxic to the yeast cells. Notably, the compounds’ inhibitory effects against the growth of the various PMT-complemented yeast varied, which can be attributed to various levels of nematode PMT proteins expression and differences in the actual physiological activity of the PMT proteins in the yeast cells.

**TABLE 5:** Growth inhibition EC_50_ concentrations of PMT inhibitors against PMT- and LacZ-complemented *pem1*Δ/*pem2*Δ S. cerevisiae

NSC	Mean growth inhibition EC_50_ (μM) ± SEM on complemented yeast						
	LacZ	HcPMT1+2	AcPMT1+2	DvPMT1+2	AsPMT	OdPMT	TcPMT
87511	NA^*a*^	2.08 ± 0.29	26.40 ± 3.45	17.05 ± 1.15	25.87 ± 1.24	57.88 ± 5.18	58.24 ± 7.00
133100	NA	18.74 ± 2.07	35.08 ± 5.01	35.73 ± 3.67	23.39 ± 1.85	36.56 ± 8.4	60.13 ± 5.85
56410	50.44 ± 13.21	1.48 ± 0.21	40.26 ± 9.42	45.64 ± 7.61	50.74 ± 3.87	37.65 ± 4.87	45.14 ± 3.97
62709	NA	3.03 ± 0.26	26.88 ± 7.63	25.56 ± 5.07	15.39 ± 2.13	19.54 ± 2.56	29.76 ± 7.61
35676	6.02 ± 0.25	5.45 ± 0.95	17.59 ± 3.72	12.93 ± 1.58	15.10 ± 0.70	3.98 ± 1.08	22.88 ± 5.00
145612	NA	8.28 ± 0.99	29.31 ± 2.54	23.00 ± 2.15	28.88 ± 4.38	32.78 ± 2.64	27.17 ± 7.70
177383	NA	6.11 ± 1.84	21.93 ± 7.31	15.36 ± 1.91	12.30 ± 1.11	19.83 ± 3.90	11.76 ± 1.43

a NA, not applicable.

### Nematode PMT inhibitors possess *in vitro* anthelmintic activity against both drug-susceptible and MDR nematodes.

Fifteen compounds that showed the highest inhibitory activity against complemented yeast (NSC133100, NSC177383, NSC87511, NSC145612, NSC35676, NSC62709, NSC12097, NSC24872, NSC99791, NSC186301, NSC320301, NSC335989, NSC56410, NSC156219, and NSC177858) were screened for *in vitro* anthelmintic activity using both a fully susceptible (TxPh-2011-S) and multiple drug-resistant (MDR; UGA MDR 2020) isolate of *H. contortus*. Two assays that measure the effects of increasing concentrations of drug on two distinct phenotypes were employed: a larval development assay (LDA) that assesses the development of nematode eggs from L1 to L3 stages (36, 37), and a larval motility assay (LMA) that assesses the movement of exsheathed L3 larvae (38).

**FIG 6:**
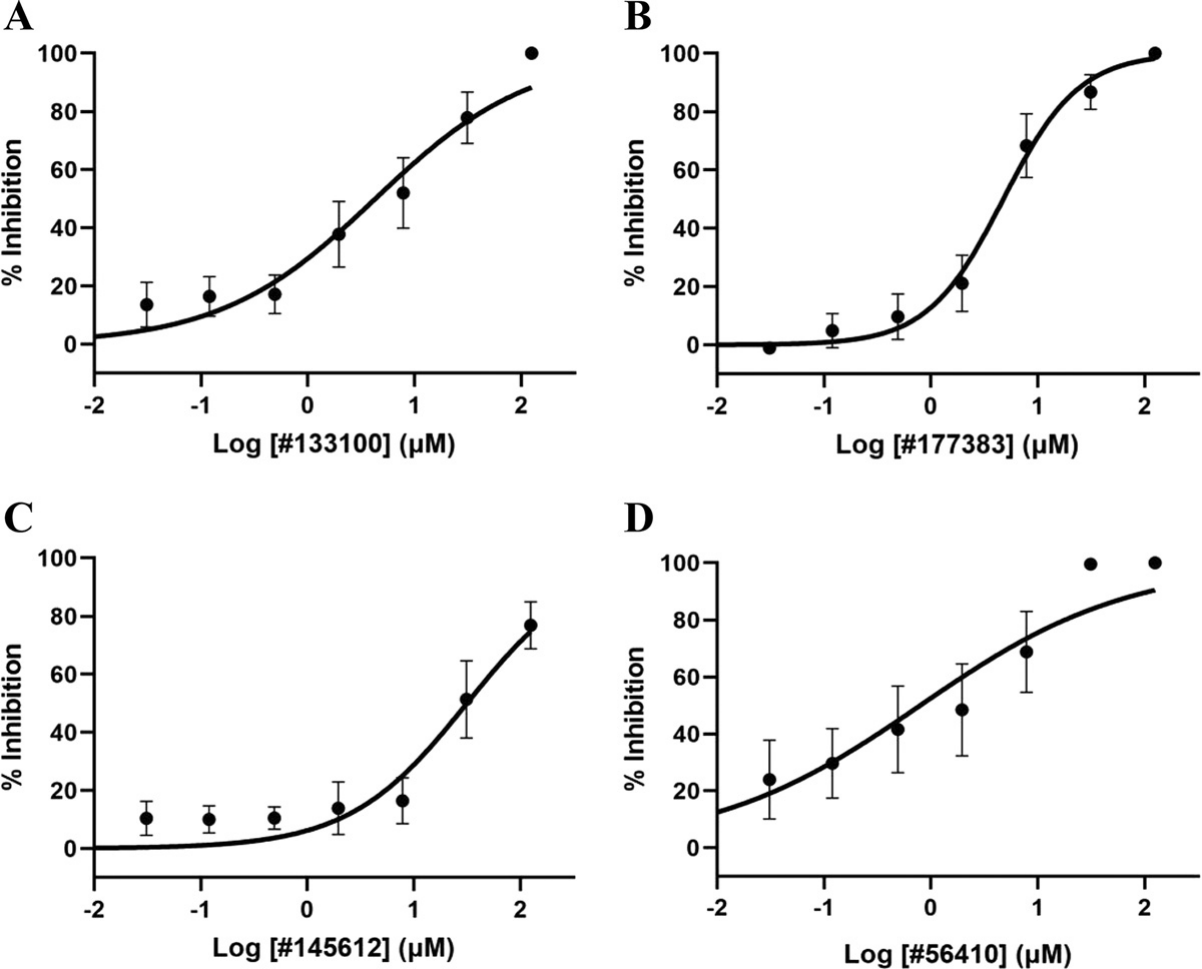
Effect of PMT inhibitors on the larval development of *Haemonchus contortus*. The percent inhibitions of larval development at various concentrations of NSC133100 (A), NSC177383 (B), NSC145612 (C), and NSC56410 (D) are depicted as dose-response curves. The data presented are averages for the compounds’ effects on both drug-susceptible and MDR isolates of *H. contortus.* Curves were fitted using a log[inhibitor] versus normalized response variable slope model in Prism 9.4.0 (GraphPad). Error bars represent the standard errors of the mean (SEM) for the mean response.

Using the LDA, 4 of the 15 compounds tested demonstrated fairly potent anthelmintic activity, with no differences in response between the MDR and drug-susceptible isolates. Dose-response curves for these compounds are shown in Fig. 6. The derived IC_50_ values with 96% confidence intervals, goodness of fit (*R*^2^) values, and hill slope values for the four compounds are shown in Table 6. One assay plate was discarded for compound NSC56410 due to poor larval development, resulting in fewer data points. Despite a high level of interassay variability, all compounds exhibited a dose response with IC_50_ values in the low micromolar range, indicating they possessed potent anthelmintic efficacy. The small differences in IC_50_ values between compounds NSC133100 and NSC177383 may not be very meaningful with regard to predictions of compound potency given the variability in the Hill slope of the dose response, but these two compounds were clearly more potent than NSC145612 by ~7-fold. On the other hand, compound NSC56410 showed the highest potency among the four compounds, with ~5-fold, 6-fold, and 39-fold higher potency than NSC133100, NSC177383, and NSC145612, respectively. Between the compared compounds, the IC_50_ values with nonoverlapping 95% CI indicated that they were statistically significantly different and vice versa.

**TABLE 6:** Anthelmintic IC_50_s for PMT inhibitors against both susceptible and MDR isolates of *H. contortus* determined by LDA

PMT inhibitor	IC_50_ (μM)^*a*^	*R* ^2^	Hill slope
NSC133100	4.29 (2.15–8.28)	0.60	0.60
NSC177383	4.76 (3.47–6.53)	0.78	1.23
NSC145612	31.49 (19.41–52.75)	0.55	0.79
NSC56410	0.80 (0.27–2.20)	0.45	0.45

a The IC_50_ was derived from dose-response curves of the percent inhibition in larval development exerted by each compound. The 95% confidence interval in indicated in parentheses.

**FIG 7:**
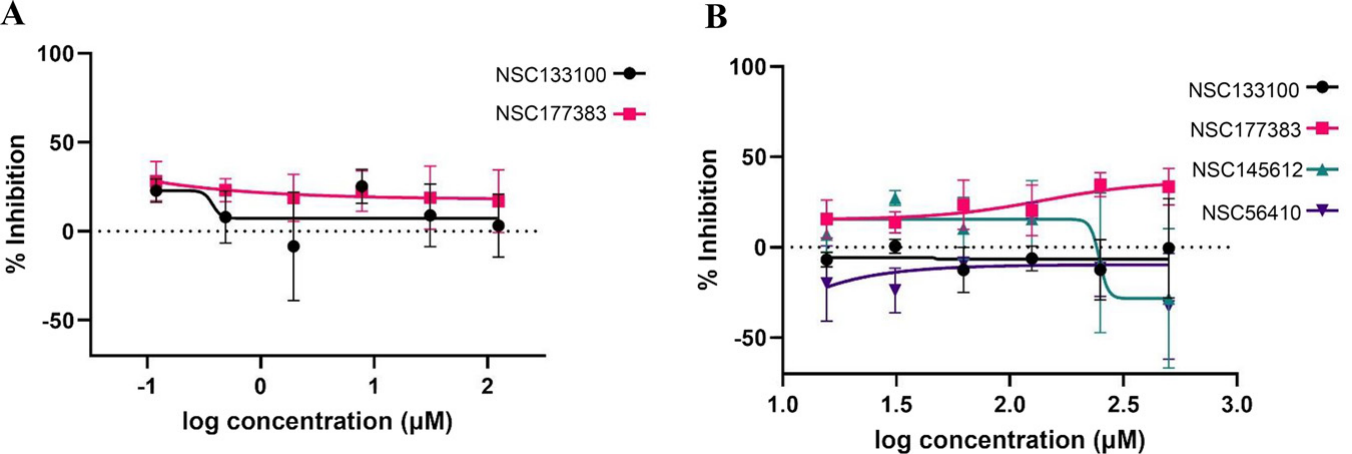
Effect of PMT inhibitors on the larval migration of *Haemonchus contortus*. The percent inhibitions of larval migration for initial lower concentrations for NSC133100 and NSC177383 (A) and extended concentrations for NSC133100, NSC177383, NSC145612, and NSC56410 (B) are depicted as dose-response curves after 24 h of treatment. Curves were fitted using a log[inhibitor] versus normalized response variable slope model in Prism 9.4.0 (GraphPad). Error bars represent the SEM for the mean response.

The LMA was also used to screen for compound activity. This assay measures motility of *H. contortus* L3 as a phenotype for detecting anthelmintic activity in candidate compounds. The LMA was performed using the Worminator system (38), a high-definition digital video imaging system that provides real-time quantitative measurement of nematode motility. LMAs were performed in parallel with the LDAs for the first two compounds tested (NSC133100 and NSC177383). Though both compounds demonstrated potent activity with the LDA (Fig. 6A and B), no anthelmintic activity was observed at any of the drug concentrations or time points tested for the LMA (Fig. 7A). To further investigate the discrepancy in results between the two assays, we performed a second round of LMAs on the four compounds (that had anthelmintic activity in the LDA assay) using 4-fold higher drug concentrations (up to 500 μM). Even with the higher concentrations, no measurable activity was seen using motility as screening phenotype (see Tables S2 to S6). Figure 7 shows the results for the 24-h reading for both rounds of testing.

## DISCUSSION

The nematode families *Trichostrongylidae*, *Dictyocaulidae*, *Chabertiidae*, *Ancylostomatoidea*, and *Ascarididae* include parasitic species of health and economic importance in humans and animals (1). For over 5 decades, the use of anthelmintic drugs has been the primary method of control for nematode infections in both humans and animals, but there is now an alarming worldwide prevalence of drug-resistant parasitic nematodes, especially in livestock (13). Furthermore, many reports have recently documented resistance to all major classes of anthelmintics (benzimidazoles, macrocyclic lactones, and tetrahydropyrimidines/imidazoles) (13). Thus, there is an urgent need to identify novel molecular targets for developing new effective anthelmintics.

Within the infected host, nematodes are prolific egg layers, requiring active biogenesis of nematode plasma membranes in which phospholipids, particularly phosphatidylcholine (PtdCho) are major constituents (19, 20). Significant disparities in fundamental biochemical and metabolic pathways of phospholipids between parasitic nematodes and their animal hosts exist. The plant-like phosphobase methylation pathway involving the three-step SAM-dependent methylation of phosphoethanolamine (PE) to phosphocholine (PCho) (catalyzed by PMT proteins) for the biosynthesis of PtdCho is unique to nematodes and essential, but it does not exist in mammals (24, 27, 28, 30–32).

In the present study, we identified, cloned, expressed, and functionally characterized the putative PMTs of parasitic nematodes from five different families of medical and veterinary importance namely, *Ancylostomatoidea*, *Ascarididae*, *Chabertiidae*, *Dictyocaulidae*, and *Trichostrongylidae*. Four PMT1 proteins containing a putative N-terminal methyltransferase domain were AcPMT1 from *A. duodenale*, a hookworm that infects humans, AsPMT from *A. suum*, a common swine parasitic nematode of economic importance, DvPMT1 from *D. viviparus*, a lungworm of cattle, and OdPMT from *O. dentatum*, a common swine nematode. Those PMT1 proteins were found to catalyze the methylation of PE to phosphomonomethylethanolamine (PMME), consistent with the enzymatic activity of previously reported orthologous PMT1 proteins in C. elegans and H. contortus (25, 29). Three putative PMT2 proteins containing the C-terminal methyltransferase domain—AcPMT2 from *A. ceylanicum*, a hookworm that infects both humans and animals; DvPMT2 from *D. viviparus*; and TcPMT2 from *T. canis*, a common nematode that infects dogs and humans—were also identified and characterized. Those PMT2 proteins were orthologous to the C. elegans and *H. contortus* PMT2 proteins known to catalyze the subsequent two-step methylation of PMME to phosphodimethylethanolamine (PDME), and eventually to PCho (21, 25, 29).

Consistent with our previous observations, the identified PMT2 proteins (AcPMT2 and DvPMT2) orthologous to the *H. contortus* HcPMT2 did not depict significant catalytic activity for the methylation of PE to PMME *in vitro* (27). However, TcPMT from *T. canis*, which is orthologous to HcPMT2, was found to possess significant catalytic activity for the methylation of PE to PMME. Notably, unlike other nematodes whose genomes contained both putative PMT1 and PMT2, *T. canis* genome yielded only a putative PMT2. X-ray crystal structures of HcPMT1 and HcPMT2 have illustrated the conserved nature of the SAM-binding fold at the catalytic domains and also revealed the structural differences at the phosphobase binding site that can be associated with the differences in the substrate preferences of the two PMT proteins (29). At the catalytic domain of HcPMT1, the phosphobase binding site is constrained by surrounding amino acids (Trp14, Met26, Met27, and Trp123) that facilitate methylation of PE to PMME but do not favor additional methylation (29). In contrast, HcPMT2 has a different collection of residues at the active site that are responsible for widening the substrate binding site so that it accommodates the larger PMME and PDME molecules (29). It is likely that TcPMT has a rearranged configuration of amino acids at the phosphobase binding site which allows both PE and the larger PMME and PDME molecules to bind and, therefore, functions like the tripartite P. falciparum PMT which has the ability to catalyze all three methylation steps (29, 39, 40). By enzyme kinetic analysis, all the identified putative PMTs, with activity for the methylation of PE, depicted Michaelis constant (*K_m_*) values for SAM that were relatively lower than those reported for C. elegans and *H. contortus* PMT1 proteins (24, 25, 28), suggesting that they possessed relatively higher affinity for the methyl-donor/cosubstrate SAM.

By complementing a mutant S. cerevisiae strain lacking the ability to endogenously synthesize PtdCho (that is essential for yeast growth in medium without exogenous choline), we validated that nematode PMTs are critical *bona fide* enzymes in the biosynthesis of PtdCho. Wild-type S. cerevisiae mainly uses the Bremer-Greenberg pathway in which phosphatidylethanolamine (PtdEA) is converted to PtdCho by two SAM-dependent phosphatidylethanolamine methyltransferases, PEMT1 and PEMT2 (33, 34, 41, 42). PEMT1 only catalyzes the conversion of PtdEA to phosphatidyl-*N*-monomethylethanolamine (PtdMME), which can then be methylated by PEMT2 to phosphatidyl-*N*,*N*-dimethylethanolamine (PtdDME), and eventually to PtdCho (33, 34, 43). Yeast uses the Kennedy pathway as an alternative route for PtdCho biosynthesis, in which exogenous choline is phosphorylated to PCho, and subsequently converted to PtdCho (19, 44, 45). In the present study, by complementing the mutant yeast (lacking PEM1 and PEM2) with the nematode PMTs, we were able to rescue the growth of the mutant through restoring the *de novo* biosynthesis of PtdCho in the absence of exogenous choline, but with ethanolamine supplementation. These findings indicate that expression of the nematode PMTs in mutant S. cerevisiae introduced the nematode phosphobase pathway through which PtdCho could be synthesized via the nematode PMT-based methylation of PEA to PCho, which then feeds into the Kennedy Pathway. Consistent with the rescued growth of the complemented mutant yeast, by using radioisotope incorporation assays, we found that radiolabeled substrate, [^14^C]ethanolamine, was incorporated into the PtdCho synthesized in the yeast.

Using an *in vitro* PMT assay, with the recombinant nematode PMT proteins as enzymes, we screened a natural compound library and identified compounds with cross-inhibitory activity against the catalytic activity of the various nematode PMTs at nontoxic concentrations. Corroboratively, we found that those PMT inhibitors blocked the growth of PMT-complemented mutant yeast by inhibiting the enzymatic activity of the nematode-derived PMTs and thereby shutting down the *de novo* biosynthesis of PtdCho via the phosphobase pathway. Collectively, these findings provide genetic and biochemical evidence for the role of the putative nematode PMTs as *bona fide* PMTs for the biosynthesis of PtdCho.

Among the identified broad-spectrum PMT inhibitors that also blocked growth of nematode PMT-complemented mutant yeast (see Fig. S6), NSC62709 is structurally similar to streptonigrin, which is an antimicrobial and antineoplastic agent isolated from Streptomyces flocculus (46). The difference between the two is that streptonigrin has a dioxo-amino-quinoline, while NSC62709 has a dimethyl-imidazole-quinoline, which likely makes NSC62709 more tolerable in mammalian cells than streptonigrin is (47). NSC35676 (see Fig. S6) is a benzotropolone-containing natural product that is contained in the nutgall of *Quercus* spp. and certain oak barks (48, 49). NSC35676 is also known as purpurogallin, and has been found to have antimicrobial and antioxidant properties (49–51). It is noteworthy that purpurogallin (NSC35676) has previously been reported to inhibit the catalytic activity of another SAM-dependent methyltransferase, catechol *O*-methyltransferase (COMT), isolated from both human and the yeast, Candida tropicalis (52, 53). These findings are consistent with the inhibitory effect of purpurogallin (NSC35676) against diverse PMT proteins in the present study. NSC145612, NSC133100, and NSC177383 (see Fig. S6) are structurally similar to rifampin and its derivatives with activity against mycobacteria (54, 55).

The anthelmintic activity of 15 PMT inhibitors was evaluated against *H. contortus* using two different screening phenotypes. The LDA evaluates the development of nematode eggs to the L3 stage and thus has the ability to detect the anthelmintic activity due to a multitude of effector mechanisms (36). In contrast, the motility of L3s may not be an appropriate phenotype to detect anthelmintic activity depending on the tissue- and stage-specific expression of the main molecular targets of the drugs assessed (56). Potent commercially available anthelmintic drugs that induce paralysis of the body wall muscle often exert a dose-response on motility (56–58). However, these responses on motility often are highly variable and only occur at high concentrations. Consequently, the motility phenotype often provides less reliable data, particularly when attempting to discriminate drug-resistant from drug-susceptible isolates (56, 58). In addition, drugs that do not directly induce paralysis, such as benzimidazoles, do not provide a useful dose-response for motility (58). Our results clearly demonstrate that the motility of the L3 stage does not constitute a useful predictor of anthelmintic activity for PMT inhibitors. This is relevant, since motility is the most common phenotype used in high-throughput screening systems (59, 60). These and other recently published data strongly indicate that the screening of experimental compounds for anthelmintic activity can be considerably improved by using *in vitro* assays targeting more than one phenotype.

Here, we show that PMT enzymes are functional and druggable targets in *H. contortus* and likely in many other nematodes that possess PMT orthologs and catalytic activity, such as *O. dentatum*, *A. suum*, and *D. viviparus*, among others. Four PMT inhibitors (NSC133100, NSC177383, NSC145612, and NSC56410) showed activity in the lower micromolar range when tested against both drug-susceptible and MDR isolates of *H. contortus*. Since targeting the enzymes in the phosphobase methylation pathway constitutes a novel mechanism of action, activity against the MDR isolate was expected to be similar to the susceptible. As expected, no differences were observed between the two isolates, further confirming this premise. LDA wells with 100% inhibition contained only larvae at the L1 stage when development was stopped at day 7. This suggests that these compounds do not possess ovicidal effect but have a mechanism of action that prevents the development of larvae once they hatch. Nematodes are a phylum of molting animals (61). Thus, by affecting membrane biogenesis, these PMT inhibitors could potentially have deleterious effects on molting, egg production by females, establishment of L4 in host tissues, host-parasite interactions, and any other biological process that requires active synthesis of phosphatidylcholine (21, 62, 63). While the effect observed in our experiments is limited to larval stages of nematodes, it is likely that PMT inhibitors would have effects against adult nematodes as well. Since phosphatidylcholine accounts for 40 to 60% of cell membrane phospholipid content (19), it influences cell membrane curvature and vesicle formation (64), which in turn is critical for overall cell functionality and survival. Further, in nematodes, changes in cell membrane composition are crucial for the parasite to efficiently adapt to variations in host animal physiological dynamics (65). This suggests that depletion of phosphatidylcholine levels in nematodes through inhibition of PMTs would lead to loss of viability and eventual death of adult worms, underscoring the potential efficacy of PMTs inhibitors against adult nematodes, in addition to their lethal effect on larval stages. Moreover, activity of commercially available anthelmintic classes in the LDA is a good predictor of drug efficacy *in vivo* (37, 66, 67). Therefore, it is reasonable to expect that the efficacy observed in the free-living stages of *Haemonchus* will translate to efficacy against adult stages.

The data presented here constitute a proof of concept and further validation of PMTs as a novel drug target in parasitic nematodes. PMT inhibitors have the potential to serve as a new generation of molecular target-specific broad-spectrum anthelmintic drugs for the treatment of nematode infections in livestock and humans.

## MATERIALS AND METHODS

### Identification of putative nematode PMTs.

Mining of nematode protein and genome databases was performed by a BLAST search using amino acid sequences of the PMTs for the parasitic nematode Haemonchus contortus that have been previously cloned and characterized as *H. contortus* phosphoethanolamine methytransferase-1 (HcPMT1) and *H. contortus* phosphoethanolamine methytransferase-2 (HcPMT2) (27, 29). The HcPMT1 N-terminal methyltransferase domains that catalyze the first step of methylating phosphoethanolamine (PE) to phosphomonomethylethanolamine (PMME) and the HcPMT2 C-terminal domains that functions to catalyze subsequent methylation steps to convert PMME to phosphodimethylethanolamine (PDME) and PDME to phosphocholine were targeted as the conserved sequences (28, 29). The retrieved sequences were analyzed by multiple sequence alignment using TCoffee (http://tcoffee.crg.cat/apps/tcoffee/do:regular).

### Cloning and expression of putative PMTs from different families of nematodes.

The coding sequences for each putative PMT were retrieved from GenBank and submitted to Integrated DNA Technologies (IDT, USA) for synthesis. The submitted putative PMT coding sequences had the following GenBank accession numbers: Ancylostoma duodenum PMT1 (AcPMT1), KN730644; Ancylostoma ceylanicum PMT2 (AcPMT2), KE125104.1; Ascaris suum PMT (AsPMT), LK871972.1; Dictyocaulus viviparus PMT1 (DvPMT1), KN716207.1; D. viviparus PMT2 (DvPMT2), KN716960.1; Oesophagostomum dentatum PMT (OdPMT), KN550380.1; and Toxocara canis PMT (TcPMT2), JPKZ01000489.1. The synthesized gene fragments were supplied by IDT cloned in pUCIDT (AMP) vector and lyophilized. Before use, they were reconstituted in sterile molecular-grade nuclease-free water. The primer pairs used for PCR-amplification of the PMT coding gene fragments for directional cloning in the pET15b expression vector (Novagen) in-frame with the N-terminal hexahistidine tag (His tag) are listed in Table S7. The recombinant expression vectors were sequenced to confirm identity of the PMT insert, amplified in the K-12 strain of E. coli cells (NEB Turbo; New England Biolabs) and transformed into protein expression BL21-CodonPlus (DE3)-RIL E. coli (Agilent Technologies). For *H. contortus* PMTs (HcPMT1 and HcPMT2), the protein expression E. coli cells generated previously were used (27). Transformed E. coli were cultured at 37°C in Luria broth medium containing 100 μg/mL ampicillin and 34 μg/mL chloramphenicol to an absorbance of 0.6 to 0.8 at a wavelength of 600 nm, and protein expression induced by addition of 1 mM isopropyl-β-d-thiogalactopyranoside (IPTG). For HcPMT1, after induction with IPTG, the culture was incubated at 16°C with shaking for 15 h. For the rest of the PMTs, bacteria were cultured at 37°C for a further 3 h after induction.

Bacterial cells were pelleted by centrifugation. For HcPMT1, 1 g of bacterial pellet was resuspended in 15 mL of lysis buffer (20 mM imidazole, 50 mM Tris base, 500 mM NaCl, 10% [vol/vol] glycerol [pH 8.0]) containing a 1× EDTA-free protease inhibitor cocktail (Pierce) and 75 kU of rLysozyme (Sigma). For the rest of the PMTs, 1 g of bacterial pellet was resuspended in 15 mL of lysis buffer composed of 50 mM NaH_2_PO_4_, 300 mM NaCl, 10 mM imidazole, 5% (vol/vol) glycerol, 0.5% (vol/vol) Sarkosyl, 1× EDTA-free protease inhibitor cocktail, and 60 kU of rLysozyme (pH 8.0). The resuspended pellets were lysed by sonication and clarified by centrifugation at 20,000 × *g* for 20 min at 4°C. The supernatant was collected and mixed with 2 mL of prewashed Ni-NTA His Bind Resin suspension (Novagen) and incubated at 4°C for 2 h with agitation. The His-tagged recombinant proteins were purified under native conditions by nickel-affinity chromatography according to the manufacturer’s instructions (Novagen). For HcPMT1, the wash buffer contained 20 mM imidazole, 50 mM Tris base, 500 mM NaCl, and 10% (vol/vol) glycerol (pH 8.0), while the elution buffer was composed of 250 mM imidazole, 50 mM Tris base, 500 mM NaCl, and 10% (vol/vol) glycerol (pH 8.0). For the rest of the PMTs, the wash buffer contained 50 mM NaH_2_PO_4_, 300 mM NaCl, and 20 mM imidazole (pH 8), while the elution buffer was composed of 50 mM NaH_2_PO_4_, 300 mM NaCl, 10 mM imidazole, 5% (vol/vol) glycerol, and 0.5% (vol/vol) Sarkosyl (pH 8). About 10 mL of the protein eluate was dialyzed in 2 L of a first buffer (50 mM Tris base, 100 mM NaCl, 10% [vol/vol] glycerol [pH 7.5]) at 4°C with stirring overnight, followed by dialysis in 2 L of a second buffer (25 mM HEPES, 100 mM NaCl, 10% [vol/vol] glycerol [pH 6.5]) at 4°C for 3 h. Further buffer exchange and concentration of the eluates was performed using protein concentrators (Fisher Scientific). The sample was first centrifuged for 20 min at 4,000 rpm at 12°C. The exchange buffer (5 mM HEPES-KOH, 0.5 mM dithiothreitol [pH 7.8]) was added to make up the volume to 20 mL, followed by centrifugation for 20 min at 1,500 × *g* at 12°C. Buffer exchange was repeated seven times, and the final sample volume was reduced to ~1 mL. The purity of the recombinant protein was analyzed by SDS-PAGE, and the concentration was measured using a Qubit 3.0 fluorometer (Life Technologies).

### Generation of PMT monospecific antisera.

The experimental animals (rats) procedures in this study were performed according to Tuskegee University Institutional Animal Care and Use Committee approved protocol R1110-18-1. Blood was collected from five rats prior to immunization and used to extract preimmune sera. To raise polyclonal antibodies against PMT proteins, the purified recombinant proteins were emulsified with Freund complete adjuvant (Sigma-Aldrich) and injected into the five rats at 40 μg of protein per rat. Two subsequent booster immunizations were administered at 2-week intervals with 20 μg of the recombinant proteins emulsified in Freund incomplete adjuvant. The rats were sacrificed, blood was collected by cardiac puncture, and sera were isolated from the blood. Pooled preimmune and pooled immune sera were used in Western blotting assays to assess the specificity of the antisera against the recombinant proteins. Purification of the PMT antisera was performed as previously described (26). Briefly, 8 mL of slurry of Affi-Gel 15 (Bio-Rad) was washed twice with 50 mL of distilled water, followed by two washes in 50 mM NaHCO_3_ buffer. The washed resin was mixed with 4 mL of solution containing 4 mg of the respective purified recombinant protein, followed by incubation at 4°C for 2 h with shaking. The mixture was centrifuged, the supernatant was removed, and the resin was washed with phosphate-buffered saline (PBS), followed by the addition of 800 μL of glycine (1 M, pH 12) and incubation at 4°C for 1 h. The mixture was washed twice in 50 mL of PBS, 4 mL of crude antiserum was added, and the mixture was incubated at 4°C with shaking overnight. The mixture was packed into a 15-mL column, and after four washes with PBS, the monospecific antibodies were eluted with 15 mL of 0.1 M glycine (pH 12.0). The eluate was collected in 1-mL fractions and neutralized with 150 μL of 1 M Tris (pH 6.8).

### *In vitro* enzyme kinetics of putative nematode PMTs.

We customized a commercial methyltransferase HT activity assay kit (ENZO) for use of recombinant PMT proteins as enzymes, *S*-adenosyl-l-methionine (SAM) as the methyl donor, and PE as the substrate, as reported previously (27). To determine whether the recombinant PMT proteins possessed methyltransferase activity, we initially tested the activity of each protein at the conditions recommended by the kit manufacturer, with SAM and PE being used at final concentrations of 100 and 200 μM, respectively. Briefly, the reaction mixture contained 2.5 μL of the kit SAM-free methyltransferase reaction buffer, 3 μL of 10 mM PE solution (pH 7.5), 15 μL of 1 mM SAM solution (pH 7.8), and 1× transferase assay buffer to a final volume of 25 μL. Various concentrations (25-μL total volume) of recombinant PMT protein were added to reactions.

To determine the enzyme kinetic parameters on SAM, fixed concentrations of PMT recombinant proteins (between 15 and 100 ng/μL, depending on the derived optimal concentration) and PE (200 μM) were used in the reactions, with various concentrations of SAM (0 to 160 μM). On the other hand, to determine the enzyme kinetic parameters on PE, fixed concentrations of PMT recombinant proteins (between 15 and 100 ng/μL, depending on the derived optimal concentration) and SAM (100 μM) were used, with various concentrations of PE (0 to 250 μM).

For normalization, instead of purified protein, 25 μL of 100 μM *S*-(5′-adenosyl)-l-homocysteine (SAH) as a positive control, 25 μL of dialysis buffer as a negative control, and 25 μL of 1× transferase assay buffer as a blank control were added to the respective reaction mixtures. The reactions were prepared in triplicate wells using black flat-bottomed 96-well plates. Detection buffer (100 μL) was added into each reaction, yielding a final reaction volume of 150 μL. The plates were covered in aluminum foil and incubated at room temperature with shaking for 30 min, and fluorescence read at 380_EX_/520_EM_ using a SpectraMax Gemini EM microplate reader (Molecular Devices). Sample readouts were normalized with positive and negative controls.

### PMT complementation of *pem1*Δ/*pem2*Δ *S. cerevisiae*.

The *pem1Δpem2Δ* S. cerevisiae mutant strain (SKY010) used in this study was generously provided by Ryouichi Fukuda (Department of Technology, University of Tokyo, Tokyo, Japan). The strain can only grow in medium supplemented with choline, and as such, was propagated in 1069 YPAD medium (ATCC) supplemented with 1 mM choline chloride (Sigma) at 30°C (35). The PMT coding gene fragments were PCR amplified using the respective primer sets listed in Table S8 and T/A-cloned into the expression vector using a pYES2.1 TOPO TA yeast expression kit (Invitrogen). The recombinant plasmids were sequenced to confirm gene identity and correct orientation. Competent *pem1Δpem2Δ* S. cerevisiae was generated with an *S.c*. EasyComp transformation kit (Invitrogen) according to the manufacturer’s instructions. The competent *pem1Δpem2Δ* S. cerevisiae was transformed with recombinant pYES2.1 plasmid harboring respective PMT-coding gene fragments or the negative-control LacZ-coding gene fragment following the instructions in the pYES2.1 TOPO TA yeast expression kit. For nematode PMTs having both PMT1 and PMT2, the yeast cells were transformed with a mixture of plasmids harboring both (HcPMT1+HcPMT2, AcPMT1+AcPMT2, or DvPMT1+DvPMT2), while those with only one PMT were transformed with a single plasmid harboring the respective PMT (AsPMT, OdPMT, or TcPMT). The pYES2.1 TOPO vector has a URA3 selection marker which enables the uracil auxotrophic *pem1Δpem2Δ* S. cerevisiae to grow in uracil-deficient medium. Therefore, uracil-deficient selection plates containing 6.7 g/L yeast nitrogen base without amino acids (Sigma), 1.92 g/L yeast synthetic dropout medium supplement without uracil, 20 g/L agar, 2% glucose, and 1 mM choline chloride were used to grow the transformed yeast colonies. Ensuing colonies were analyzed by PCR to confirm presence of recombinant pYES2.1 plasmid bearing the respective PMT coding sequence. Positive colonies were inoculated and propagated in 15 mL of selection medium (6.7 g/L yeast nitrogen base without amino acids, 1.92 g/L yeast synthetic dropout medium supplement without uracil, 2% glucose, and 1 mM choline chloride) at 30°C with shaking at 275 rpm overnight.

To determine the ability of PMT- or LacZ-complemented *pem1Δpem2Δ* S. cerevisiae to grow in the absence of choline but in the presence of ethanolamine, 150 μL of the overnight culture was pelleted by centrifugation and resuspended in 10 mL of fresh minimal medium (containing 3.4 g/L yeast nitrogen base without amino acids, 2% glucose, 30 mg/L histidine, 100 mg/L leucine, 100 mg/L adenine and 50 mg/L tryptophan without choline) and cultured at 30°C with shaking for 24 h to starve the cells of choline. The cells were pelleted and resuspended in 1 mL of fresh minimal medium (containing 3.4 g/L yeast nitrogen base without amino acids, 2% galactose, 30 mg/L histidine, 100 mg/L leucine, 100 mg/L adenine, and 50 mg/L tryptophan) and 900 μL of the suspension further diluted 10 times with the same medium, with or without 1 mM ethanolamine. The diluted cultures were incubated at 30°C with shaking for 4 days, during which time the optical density at 600 nm (OD_600_) was measured every 24 h using a SpectraMax Plus 384 microplate reader (Molecular Devices) to monitor growth.

### Western blotting.

To determine the expression of PMTs in complemented yeast, 2 mL of the cultured yeast (with the opacity density adjusted to equal amounts) induced for transgene expression were pelleted and resuspended in 1 mL of PBS. The cells were lysed by repeated freeze-thaw, followed by sonication and the addition of an equal volume of Laemmli sample buffer. The samples were boiled for 5 min and snap-chilled on ice, followed by centrifugation. For each sample, 30 μL of the cleared lysate was fractionated by SDS-PAGE and transferred onto a nitrocellulose membrane. Immunoblotting was done using the respective monospecific-purified rat antisera at a dilution of 1:400 as primary antibodies, and horseradish peroxidase-conjugated chicken anti-rat (Thermo Fisher Scientific) as the secondary antibody at a 1:2,000 dilution. Signal generation was performed using Clarity Western ECL substrate (Bio-Rad), and imaging was performed using a FluoroChem R imager (Protein Simple).

### Radioisotope incorporation, lipid extraction, and 2D-TLC.

After choline starvation (as described above), the complemented *pem1*Δ/*pem2*Δ S. cerevisiae cultures were diluted 10× and cultured in minimal medium (3.4 g/L yeast nitrogen base without amino acids, 2% galactose, 30 mg/L histidine, 100 mg/L leucine, 100 mg/L adenine, and 50 mg/L tryptophan) containing 1 mM ethanolamine hydrochloride and 0.8 μCi/mL [^14^C]ethanolamine hydrochloride (American Radiolabeled Chemicals, USA). Due to the inability of the LacZ-complemented *pem1*Δ/*pem2*Δ S. cerevisiae to grow in the absence of choline, 1 mM choline chloride was added to its culture. The cultures were incubated for 72 h at 30°C with shaking, after which the OD_600_ of cultures was adjusted to the same level by diluting with an appropriate volume of medium. From each culture, 3 mL was harvested by centrifugation, and the pellet was washed in 10 mL of PBS twice. The lipids were extracted as reported previously (68), with some modifications. Briefly, the washed cell pellet was resuspended in 20 volumes of chloroform-methanol solvent (2:1, vol/vol), and the cells were lysed by sonication. The samples were then agitated in a shaker for 20 min at room temperature and the supernatant collected by centrifugation at 4,000 rpm for 15 min at 4°C. About 0.2 volumes of 0.9% NaCl solution was added to the supernatant and vortexed for 10 s, followed by centrifugation for 5 min at 2,000 rpm at room temperature to separate the aqueous (top clear layer) and organic (bottom layer) phases. The two layers were separately pipetted into sterile clean 10-mL clean glass vials and kept open in a clean bench drawer until completely evaporated. The evaporated organic phase was reconstituted in 200 μL of chloroform-methanol solvent (2:1, vol/vol). Glass chambers were conditioned by placing a 25 cm × 25 cm blotting paper vertically standing, with one end dipping in about 100 mL of first dimension solvent (chloroform-methanol-ammonium hydroxide [84.5/45/6.5, vol/vol/vol]), with the lid tightly sealed with a lining of grease overnight. The following day, Silica Gel-60 20 × 20 plates (Sigma-Aldrich) were baked at 325°C for 10 min. After cooling to room temperature, 50 μL of sample (reconstituted organic lipid phase) was spotted onto a premarked application point at one corner, about 4 cm from the edge and from the bottom. Once the spotted sample had air-dried, the plate was placed in the preconditioned chamber vertically to dip into the first-dimension solvent to a depth of about 1 cm (with the sample application just above the solvent line). The chamber was tightly sealed and the solvent left to migrate up to about 3 cm below the top edge of the plate, after which the plate was air dried and placed in the chamber with the second solvent (chloroform-acetic acid-methanol-water [90/30/6/2.6, vol/vol/vol/vol]) at 90° angle to the first direction, with the sample application point just above the solvent line, but on the other end. The plate was left for the second solvent front to migrate up to about 4 cm from the top of the plates. After air-drying, the plate was placed in an imaging cassette (with the silica side facing up), and an X-ray film (Hyperfilm ECL; Cytiva) was placed on top. The cassette was closed tightly and kept at −80°C for 2 weeks; the film was then developed for signal visualization.

### Screening of a natural compound library for PMT inhibitors.

The chemical compounds were obtained from the National Cancer Institute/Developmental Therapeutics Program Open Chemical Repository. The library consisted of 419 compounds categorized as Natural Products Set IV (see Table S1). Compounds were individually reconstituted in molecular biology grade dimethyl sulfoxide (DMSO) from Sigma-Aldrich to a stock concentration of 10 mM and stored at −20°C until use. Initial screening was conducted using recombinant HcPMT1 protein as enzyme in the methyltransferase assay, with test compounds at a final concentration of 40 μM. The test reaction mixtures consisted of 40 μM test compound, 2.5 μL of SAM-free methyltransferase reaction buffer, 150 μM PE (pH 7.5), 120 μM SAM (pH 7.8), and 20 ng/μL of HcPMT1 protein, and the final volume made up to 50 μL with 1× transferase assay buffer. The volume of each test compound added did not exceed 1% of the total reaction volume. To the positive reaction mixture, instead of test compound, an equivalent volume of DMSO was added. In the negative-control reaction mixture, instead of HcPMT1 and test compound, equivalent volumes of 1× transferase buffer and DMSO, respectively, were added. Detection buffer (100 μL) containing fluorescence substrate was added into each reaction mixture. The reactions were prepared in triplicate wells in 96-well plates. The plates were covered in aluminum foil and incubated at room temperature with agitation for 30 min, followed by reading the fluorescence at 380_EX_/520_EM_. The mean percent inhibition (MPI) of the test compound on enzymatic activity was calculated using the following formula:

$$\text{MPI}=\frac{\Delta {\text{RFU}}_{\text{positive control}}\;-\;\Delta {\text{RFU}}_{\text{compounds}}}{\Delta {\text{RFU}}_{\text{positive control}}}\times 100,$$

where the positive control is a reaction without compound but with recombinant PMT protein and a volume of DMSO equivalent to that of compound used in test reactions; the negative control is the reaction without protein and compounds, but with volumes of dialysis buffer and DMSO equivalent to protein and compound added in the test reaction mixtures, respectively; ΔRFU_positive control_ represents the absolute relative fluorescence units (RFU) for the positive control, which equals RFU_positive control_ – RFU_negative control_; and ΔRFU_compounds_ represents the absolute RFU value for the sample with the compounds, which equals RFU_compounds_ – RFU_negative control_.

### *In vitro* compound cytotoxicity assays.

Compounds were tested for cytotoxicity in human ileocecal colorectal adenocarcinoma cells (HCT-8) (ATCC CCL-244; RRID:CVCL_2478) by using the cell proliferation reagent WST-1 (Roche) according to the manufacturer’s protocol. The WST-1 assay is a quantitative colorimetric assay for measurement of metabolically active cells. This assay is based on the reduction of the tetrazolium salt (WST-1) by viable cells. About 5 × 10^4^ HCT-8 cells were seeded per well in 96-well plates and grown overnight in 200 μL of RPMI 1640 medium (without phenol red; Gibco) supplemented with 2.5 g/L of glucose, 1 mM sodium pyruvate, 1.5 g/L of sodium bicarbonate, 10% heat-inactivated FBS (Gibco), and 1× antibiotic-antimycotic (Gibco) at 37°C with 5% CO_2_ in a humidified incubator. Upon reaching 80 to 90% confluence, cells were treated with various concentrations of the test compounds for 24 h. Control wells received equivalent volumes of DMSO used in the reconstituted compounds. After 24 h of culture, 10 μL of the WST-1 reagent was added to each well, and the plates were incubated in the dark for 1 h at 37°C with 5% CO_2_. The plates were well agitated, and 150 μL of the medium from each well was transferred to a new clear flat-bottomed black 96-well plate (Corning). The absorbance was read at a test wavelength of 440 nm and a reference wavelength of 690 nm using a multimode microplate reader (Spectra Max iD3; Molecular Devices, USA). All of the reactions were prepared in triplicate. The dose-response curves were generated by nonlinear regression analysis with GraphPad PRISM software using control-normalized data to determine the half-maximal cytotoxicity (IC_50_) concentrations.

### Screening HcPMT1 inhibitors for cross-inhibitory effect against AcPMT1, AsPMT, DvPMT1, OdPMT, and TcPMT.

Compounds that had shown more than 50% inhibition on HcPMT1 activity and had no or very low cytotoxicity against HCT-8 cells were further tested for cross-inhibitory effect against the enzymatic activity of other recombinant PMT proteins (AcPMT1, AsPMT, DvPMT1, OdPMT, and TcPMT) at 40 μM. The enzymatic assay was set up as described above, with the respective PMT protein as enzyme. Compounds that depicted cross-inhibitory activity were further tested at various concentrations to derive their half-maximal effective concentrations (EC_50_) using nonlinear regression analysis by GraphPad Prism software.

### Effect of PMT inhibitors on the growth of PMT-complemented *pem1*Δ/*pem2*Δ *S. cerevisiae*.

To determine the effect of PMT inhibitors on the growth of PMT- and LacZ-complemented *pem1Δpem2Δ* S. cerevisiae, the yeast cells were first starved of choline as described above, and the cells were diluted to an OD_600_ of ~0.6. The PMT-complemented yeast cells were cultured in SG medium (3.4g/L yeast nitrogen base without amino acids, 2% galactose, 30 mg/L histidine, 100 mg/L leucine, 100 mg/L adenine, and 50 mg/L tryptophan) supplemented with 1 mM ethanolamine hydrochloride, while the LacZ-complemented yeast cells were cultured in SG medium supplemented with 1 mM choline chloride. Compounds that depicted cross-inhibitory activity against PMT proteins *in vitro* were added at various concentrations to the cultures. As a negative control, DMSO (at a final concentration not exceeding 2% [vol/vol]) was added to yeast cultures designated control cultures. The cultures were incubated at 30°C with shaking at 275 rpm for 4 days. On a daily basis during the course of culture, 200-μL portions of culture aliquots were taken, and their OD_600_s were measured using a microplate reader. In preliminary work, we had observed that the growth of PMT- and LacZ-complemented *pem1Δpem2Δ* S. cerevisiae plateaued at 3 to 4 days of culture. Therefore, to determine the inhibitory effect of nematode PMT inhibitors on the growth of PMT- and LacZ-complemented *pem1Δpem2Δ* S. cerevisiae, measurements taken on day 3 were used. The results were presented as percentage of growth rate using the following formula:

$$\text{PGR = }\frac{\text{growth rate (plus inhibitor)}}{\text{growth rate (minus inhibitor)}}\times 100$$

where the percent growth rate (PGR) is the growth rate of inhibitor-treated PMT- or LacZ- complemented *Pem1Δpem2Δ* S. cerevisiae, considering the growth rate of untreated PMT- or LacZ- complemented *Pem1Δpem2Δ* S. cerevisiae is 100%; the growth rate = Δ*A*_600_^Day 3^/Δ*A*_600_^Day 0^; Δ*A*_600_^Day 0^ is the absolute value of *A*_600_ of PMT- or LacZ- complemented *pem1*Δ/*pem2*Δ S. cerevisiae at day 0, which equals the *A*_600_ of the culture minus the *A*_600_ of the growth medium only; Δ*A*_600_^Day 3^ is the absolute value of the *A*_600_ of PMT- or LacZ complemented *pem1*Δ/*pem2*Δ S. cerevisiae at day 3, which equals the *A*_600_ of the culture at day 3 minus the *A*_600_ of growth medium only; the growth rate (plus inhibitor) is the growth rate of PMT- or -LacZ complemented *pem1Δpem2Δ* S. cerevisiae in the presence of test compound; and the growth rate (minus inhibitor) is the growth rate of the PMT- or LacZ complemented *pem1*Δ/*pem2*Δ S. cerevisiae in the absence of test compound, but with a volume of DMSO equal to that of compound added in the treated culture. The dose-response curves were generated with GraphPad Prism software and IC_50_ concentrations of the compounds in PMT- and LacZ-complemented yeast cells derived.

### *In vitro* testing of anthelmintic activity of broad-spectrum PMT inhibitors.

The *H. contortus* isolates used were TxPh-2011-S (Texas A&M University, USA) and UGA MDR 2020 (University of Georgia Athens, USA). TxPh-2011-S (Texas A&M University, USA) was originally isolated from Pronghorn antelope in West Texas in 2011 and is fully drug susceptible, phenotypically determined by the DrenchRite larval development assay (LDA; Microbial Screening Technologies, Kemps Creek, Australia). To establish this as a laboratory isolate, it was initially passaged in goats at Texas A&M University and then at The University of Georgia using both goats and sheep, and it is currently maintained in lambs at Louisiana State University. A nemabiome assay (69) was conducted at the University of Calgary to confirm that the isolate is 100% *H. contortus*. The UGA MDR 2020 (University of Georgia Athens, USA) isolate is resistant to ivermectin, benzimidazoles, levamisole, and moxidectin, as determined by the DrenchRite LDA. To establish this as a laboratory isolate, feces collected from a sheep farm in Georgia with known history of multiple anthelmintic resistance were cultured. Two donor goats were infected with the larvae recovered, which were morphologically (70) identified as 89% *H. contortus.* A fecal egg count reduction test (FECRT) was then performed in the infected goats. First, a combination treatment with albendazole at 15 mg/kg and ivermectin at 0.4 mg/kg was performed, followed by a treatment with levamisole at 12 mg/kg. Later, and to confirm resistance to moxidectin, another treatment was administered using a dose of 0.4 mg/kg of moxidectin. Posttreatment feces were recovered, coprocultures were established, and L3 larvae were identified as 100% *H. contortus*. Resistance to all drugs was subsequently confirmed using a DrenchRite assay.

Nematode eggs were isolated as previously described (71). After the eggs were resuspended in water, a cleaning protocol (72) was followed to minimize contamination in the assay plates. Eggs were agitated gently in a solution of 8.4-mg/L sodium hypochlorite for 12 min and then washed three times with water. The eggs were diluted in distilled water at a concentration of 50 to 100 eggs per 20 μL after the addition of amphotericin B (final concentration, 90 μL/mL) and used immediately for LDAs.

Testing with the LDA was done in two phases. In the first phase, all compounds were tested against the susceptible isolate. In the second phase, all compounds that demonstrated activity in the first phase were retested against both susceptible and the resistant isolates, yielding between 3 and 4 biological replicate assays for each hit compound. Technical replicates were defined as replicate wells within the same assay plate with eggs derived from the same pool of feces and tested with drug solutions made from the same stock solution. Biological replicates were represented by different fecal collections and respective egg isolation on a different day from other biological replicates. Compounds dissolved in 100% DMSO were diluted in the assay plate using water as an initial diluent to 5% DMSO and then in agar to a final concentration of 1% DMSO. No precipitation was observed in any of the diluted concentrations. Eight drug concentrations were tested—125.000, 31.250, 7.810, 1.950, 0.490, 0.120, 0.031, and 0.008 μM—with three replicate wells for each concentration per plate. In the case of compound NSC99791, the original stock concentration differed from the other compounds, yielding final concentrations of 100.000, 25.000, 6.250, 1.560, 0.390, 0.098, 0.024, and 0.006 μM. Three compounds were tested in each assay plate, and 24 wells served as controls (i.e., no drug and 1% DMSO in 2% agar). Plates were prepared in advance and stored at 4°C for periods no longer than 5 days. Prior to use, plates were warmed to room temperature, and 20 μL of water was added to each well to fully hydrate the agar. Between 50 and 100 eggs contained in 20 μL of water were then added, depending on the number of eggs isolated. Plates were sealed with Parafilm to reduce moisture loss and placed in an incubator at 25°C. After 24 h, 20 μL of nutritive media composed of 0.87% Earle’s balanced salts (Sigma-Aldrich, St. Louis, MO), 1% yeast extract (BD Difco, VWR; Becton Dickinson, Sparks, MD), 0.76% NaCl (Sigma-Aldrich), and 1% E. coli OP50 was added to each well. The plates were resealed and incubated for 6 additional days, and the assays were ended with the addition of 20 μL of 50% Lugol iodine to each well. The contents of every well were then transferred to a clean 96-flat well plate, and all eggs and larvae in each well were counted under an inverted microscope as previously described (73). Development to the L3 stage was corrected for all of the treatment wells based on the average development in the control wells. The percent larval development (to the L3 stage) was calculated for each well by dividing the number of L3 by the total count of larvae (L1+L2+L3) for that well and then multiplying that value by 100. The percent inhibition in larval development (%ILD) for each individual drug well was then calculated compared to the percent development in control wells by dividing the percent larval development of each drug well by the mean percent larval development of the control wells (for that plate) according to the following formula:

$$\text{\%ILD}=\frac{{\text{mean \% development}}_{\text{control}}{\;-\;\text{mean \% development}}_{\text{treated}}}{{\text{mean \% development}}_{\text{control}}}\times 100$$

Data from wells were discarded before Prism analyses if there was evidence of excessive dryness or fungal or bacterial overgrowth. Outliers were identified by using the Z-score outlier detection method. Moreover, Z-score of zero represents a value that equals the mean while a Z-score of 2 indicates that an observation is two standard deviations above or below the mean. Any value outside the Z-score of ±2 for each given concentration was classified as outlier and discarded from further analysis. The statistical analysis of the data was done using GraphPad Prism version 9.4.0. A nonlinear regression model with a variable slope (GraphPad Software, La Jolla, CA) was the method applied. Between 9 and 12 replicate values were obtained for each drug concentration, previously corrected for control. Drug concentrations were log_10_ transformed, and then IC_50_ concentrations and dose-response curves were obtained from the log[inhibitor] versus the normalized response variable slope logistic equation for each drug.

For LMAs, the first phase testing using compounds NSC133100 and NSC177383 was done using the Worminator system in parallel with the LDA. Initial stock solutions of 12.5 mM were prepared for each compound using 100% DMSO as a solvent. Initial stock solutions (25 μL) were then diluted in 475 μL of culture media to yield working stock solutions of 625.00, 156.30, 39.10, 9.80, 2.40, 0.60, 0.20, 0.04, and 0.00 μM in 5% DMSO for each drug. In plate, 200 μL of working stock solutions were then diluted in 800 μL of culture media, yielding concentrations of 125.00, 31.25, 7.81, 1.95, 0.49, 0.12, and 0.00 μM in 1% DMSO. For the second phase of testing, compounds showing anthelmintic activity with the LDA were tested in a second round at higher concentrations than in the first phase of testing. Initial stocks of 50 mM were prepared for compounds NSC133100, NSC177383, NSC145612, and NSC56410 using 100% DMSO as a solvent. Initial stock solutions (25-μL portions) were then diluted in 475 μL of culture media consisting of 65% Lauria-Bertani (LB) (Thermo Fisher Scientific, Branchburg, NJ), 25% NCTC (Sigma-Aldrich), 10% fetal bovine serum (Atlanta Biologicals, Flowery Branch, GA), Pen/Strep (Sigma-Aldrich) at a final concentration of 100 mg/mL, and amphotericin B (Sigma-Aldrich) at a final concentration of 2.5 mg/mL. The working stock solutions yielded concentrations of 2,500.00, 625.00, 156.30, 39.10, 9.80, 2.40, and 0.00 μM in 5% DMSO for each compound. In each well in the plate, 200 μL of working stock solutions were then diluted in 800 μL of culture media, yielding final concentrations of 500.00, 125.00, 31.30, 7.81, 1.95, 0.49, and 0.00 μM in 1% DMSO.

For the motility assays, 24-well plates with inserts were used (Costar, catalog no. 3379; Corning, Inc., Corning, NY). Portions (640 μL) of culture media were added to each well, and approximately 50 exsheathed larvae contained in 160 μL of culture media in each insert. Once pretreatment readings were taken, 160 μL of the drug solution was added to the well, and another 40 μL was added to the insert, reaching the final concentrations described in the protocol above. All concentrations were tested in triplicate. After addition of the drugs, plates were incubated at 5% CO_2_ and 37°C. Motility readings were taken at 1, 4, 24, 48, 72, and 96 h. A medium change was conducted at 48 h, with new drug added. Prior to each reading, plates were shaken for 15 min at ambient temperature using a benchtop shaker to stimulate motility of the L3 larvae (previously tested in our lab). The Worminator assay was performed as previously described (56). The percent inhibition in motility for each drug well was calculated by comparing the mean motility to the mean motility of the control wells. Statistical analyses were performed using Prism 9.4.0 (GraphPad). Drug concentrations were log_10_ transformed, and a variable slope nonlinear regression algorithm was applied to the data. IC_50_ values were generated using log[inhibitor] versus response variable slope (four parameters).

### Statistical analysis.

Data were analyzed using two-way analysis of variance with Tukey’s multiple-comparison *post hoc* test using GraphPad Prism v9.4.0. In addition, Student *t* tests were performed to evaluate differences between groups. *P* values of ≤0.05 were considered significant.
