# Supplementary material for: Broad-Spectrum Inhibitors for Conserved Unique Phosphoethanolamine Methyltransferases in Parasitic Nematodes Possess Anthelmintic Efficacy
Source: Antimicrob Agents Chemother. 2023 May 22;67(6):e00008-23. doi: 10.1128/aac.00008-23 (PMC10269165; doi:10.1128/aac.00008-23)
Supplement: Supplemental file 1 — Supplemental material. Download aac.00008-23-s0001.pdf, PDF file, 1.1 MB [file aac.00008-23-s0001.pdf]

S1 Figure

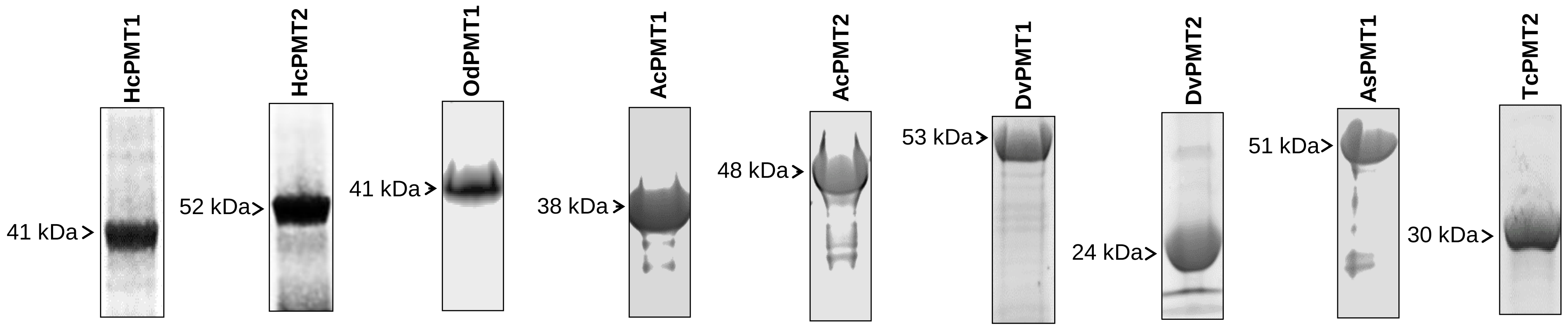

S1 Figure. SDS-PAGE analysis of the nickel affinity-purified recombinant PMT proteins. The names of the respective PMTs are indicated and their molecular weight in kDa based on the protein ladder marker. HcPMT: *Haemonchus contortus* PMT; OdPMT: *Oesophagostomum dentatum* PMT; AcPMT: *Ancylostoma ceylanicum* PMT; DvPMT: *Dictyocaulus viviparus* PMT; AsPMT: *Ascaris suum* PMT; TcPMT: *Toxocara canis* PMT.

S2 Figure

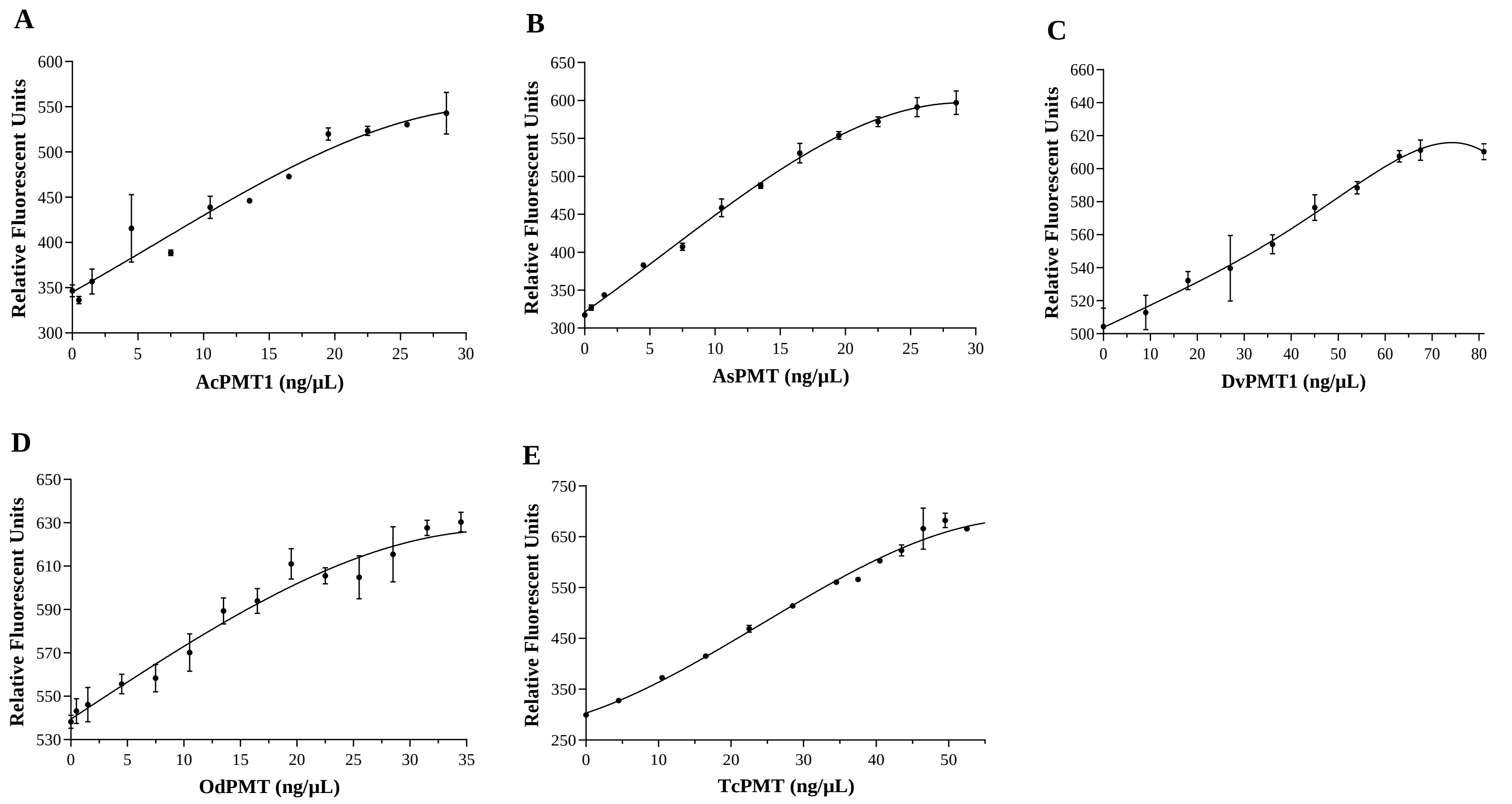

S2 Figure. Analysis of the concentration-dependent enzymatic activity of natively purified recombinant PMT proteins in the methyltransferase assay with *S*-adenosylmethionine and phosphoethanolamine each maintained at 100 μM and 200 μM, respectively. Titration curves for (A) AcPMT1, (B) AsPMT, (C) DvPMT1, (D) OdPMT, and (E) TcPMT. The data shown represent means of three independent experiments with standard error bars.

S3 Figure

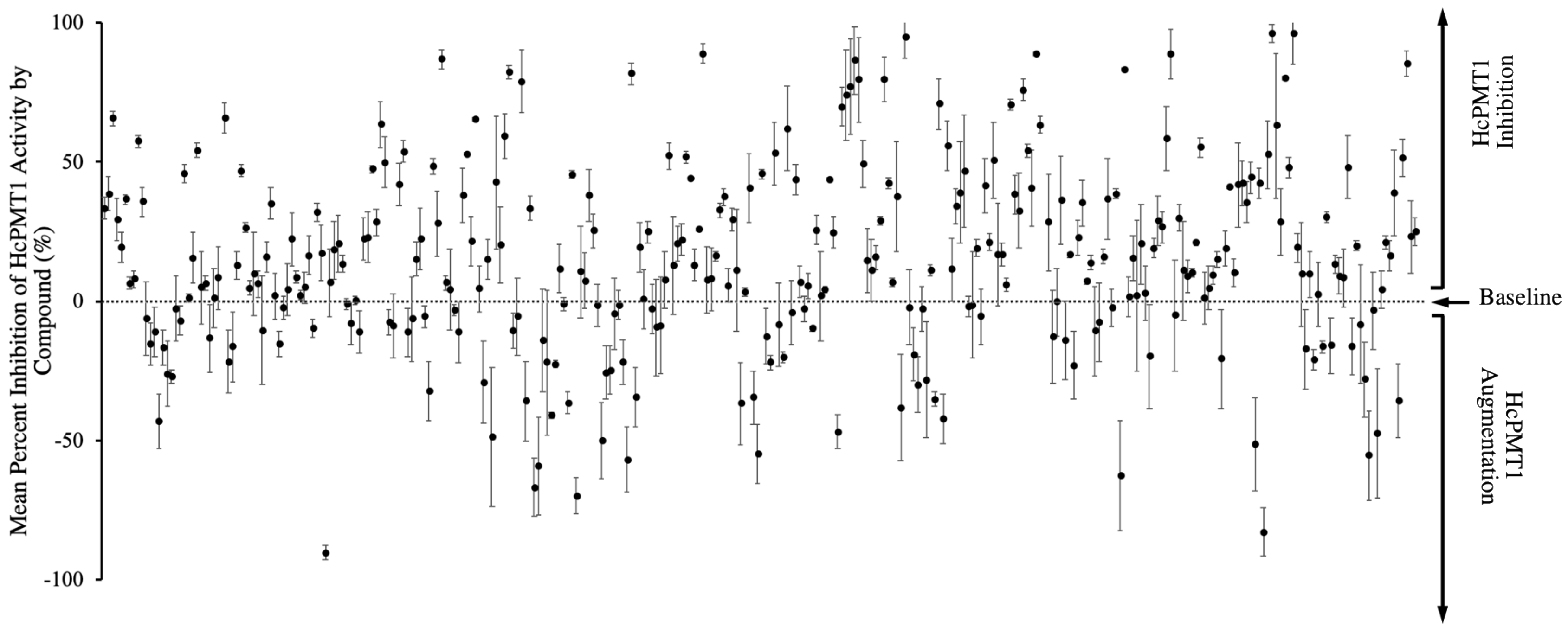

S3 Figure. Effect of compounds from the NCI Natural Product Set IV library on the enzymatic activity of *Haemonchus contortus* PMT (HcPMT1) recombinant protein. Individually reconstituted compounds were used at a final concentration of 40 μM in the HcPMT1-catalyzed reaction for the methylation of phosphoethanolamine. The mean percent inhibition of HcPMT1 activity by each compound was derived by dividing the difference in fluorescence between the compound-treated wells and the DMSO-treated wells by the fluorescence of the DMSO-treated wells and multiplying the product by 100. The baseline mean percent inhibition of 0 was for the reaction without compound, but with an equivalent volume of DMSO used to reconstitute the compounds. Compounds with mean percent inhibition values greater than 0 were designated as inhibitors of the activity of HcPMT1, while those with mean percent inhibition values less than 0 were classified as augmenters. Each reaction was performed in triplicate, and the data shown represent the mean of three independent experiments. Bars represent standard errors of the mean (SEM).

S4 Figure

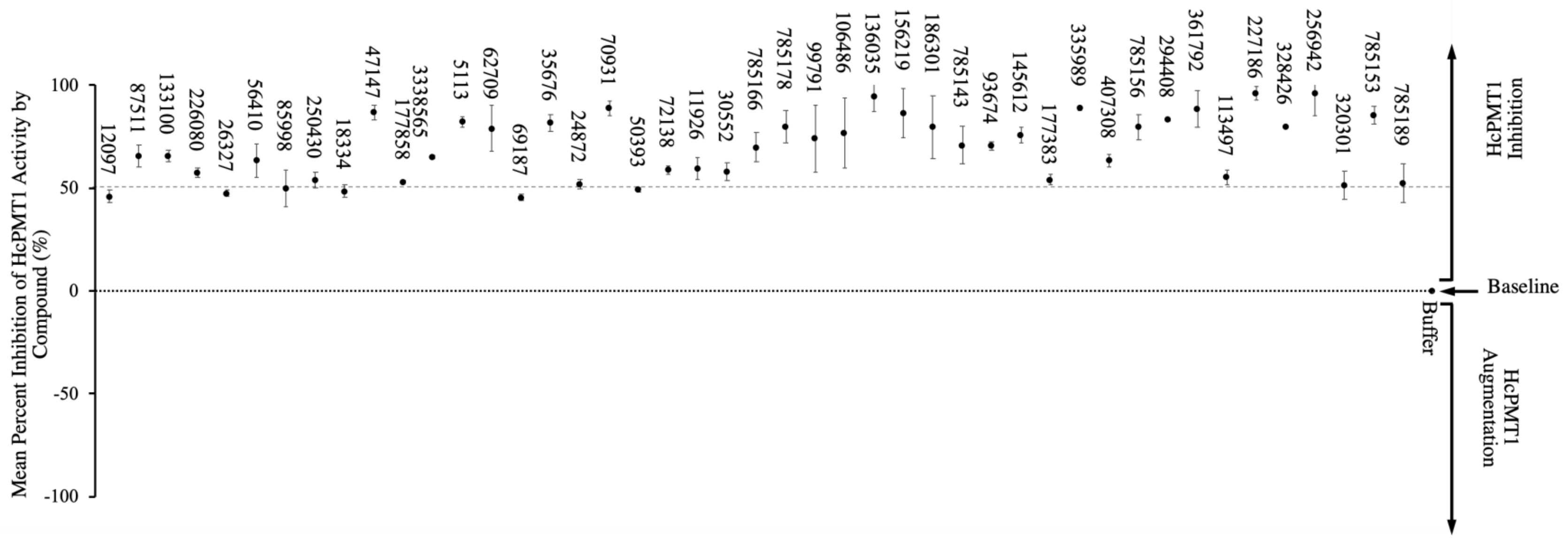

S4 Figure. Compounds from the NCI Natural Product Set IV library with >50% inhibitory activity against HcPMT1 enzymatic activity. Individually reconstituted compounds (indicated by their NSC numbers) were used at a final concentration of 40 μM in the HcPMT1-catalyzed reaction for the methylation of phosphoethanolamine. The mean percent inhibition of HcPMT1 activity by each compound was derived by dividing the difference in fluorescence between the compound-treated wells and the DMSO-treated wells by the fluorescence of the DMSO-treated wells and multiplying the product by 100. The baseline mean percent inhibition of 0 was for the reaction without compound, but with an equivalent volume of DMSO used to reconstitute the compounds. Each reaction was performed in triplicate, and the data shown represent the mean of three independent experiments. Bars represent standard errors of the mean (SEM).

S5 Figure

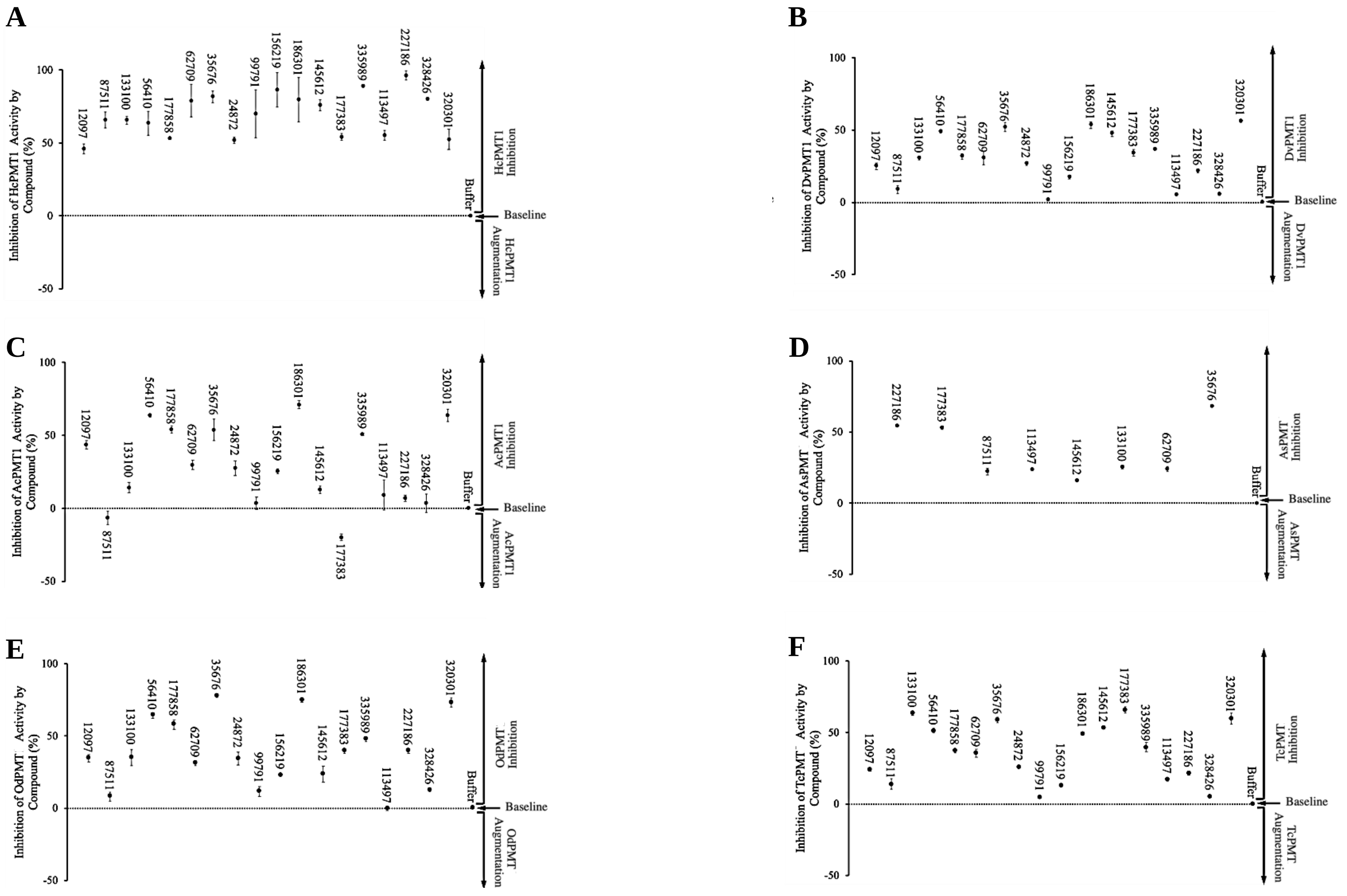

S5 Figure. Compounds from the NCI Natural Product Set IV library depicting cross-inhibitory effective against the enzymatic activity of various nematode PMTs recombinant proteins *in vitro*. Individually reconstituted compounds (indicated by their NSC numbers) were used at a final concentration of 40 µM in (A) HcPMT1, (B) DvPMT1, (C) AcPMT1, (D) AsPMT, (E) OdPMT or (F) TcPMT catalyzed reactions for the methylation of phosphoethanolamine. The mean percent inhibition of enzyme activity by each compound was derived by dividing the difference in fluorescence between the compound-treated wells and the DMSO-treated wells by the fluorescence of the DMSO-treated wells and multiplying the product by 100. The baseline mean percent inhibition of 0 was for the reaction without compound, but with an equivalent volume of DMSO used to reconstitute the compounds. Each reaction was performed in triplicate, and the data shown represent the mean of three independent experiments. Bars represent standard errors of the mean (SEM).

S6 Figure. Chemical structures of broad-spectrum nematode PMT inhibitors.

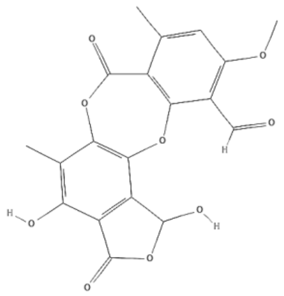

NSC87511

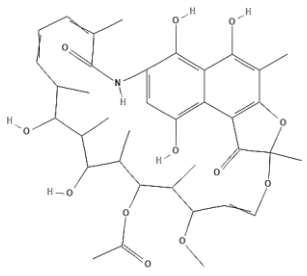

NSC133100

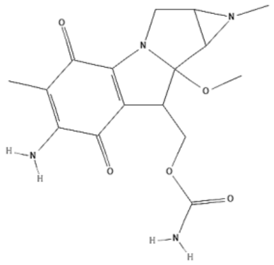

NSC56410

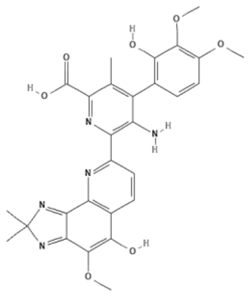

NSC62709

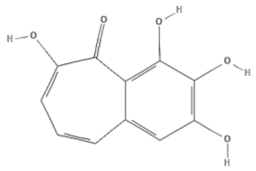

NSC35676

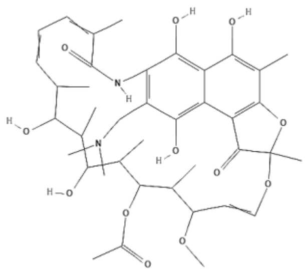

NSC145612

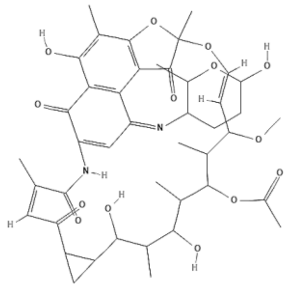

NSC177383

S1 Table. Natural Products Set IV compound library

PLATE NUMBER	WELL NUMBER	NSC NUMBER	MOLECULAR WEIGHT	MOLECULAR FORMULA
13160330	C03	757	399	C22H25NO6
13160330	C04	12097	213	C12H7NO3
13160330	C05	31048	611	C28H34O15
13160330	C06	56464	646	C34H47NO11
13160330	C07	87511	386	C19H14O9
13160330	C08	133100	721	C37H47NO12.Na
13160330	C09	226080	914	C51H79NO13
13160330	C10	302289	232	C15H20O2
13160330	C11	349438	168	C9H12O3
13160330	C12	637086	278	C13H18N4O3
13160330	C13	3716	116	C5H8O3
13160330	C14	7533	985	C49H76O20
13160330	C15	9699	270	C18H22O2
13160330	C16	15624	382	C22H26N2O4
13160330	C17	22939	224	C12H20N2O2
13160330	C18	31754	256	C14H8O5
13160330	C19	36407	414	C22H22O8
13160330	C20	46709	131	C6H13NO2
13160330	C21	62786	183	C9H13NO3
13160330	C22	76627	912	C49H85NO14
13160330	D03	7524	674	C36H51NO11
13160330	D04	23969	346	C22H35NO2
13160330	D05	36351	339	C20H21NO4
13160330	D06	71795	246	C17H14N2
13160330	D07	118343	236	C12H12O5
13160330	D08	176503	362	C20H26O6
13160330	D09	284200	345	C21H44O3
13160330	D10	332598	626	C35H47NO9
13160330	D11	382796	552	C31H25N3O7
13160330	D12	2150	152	C8H8O3
13160330	D13	5897	322	C19H14O5
13160330	D14	8661	286	C16H14O5
13160330	D15	13123	113	C4H7N3O
13160330	D16	19509	391	C21H26N2O3.ClH
13160330	D17	26254	194	C7H14O6
13160330	D18	34552	185	C10H19NO2
13160330	D19	43338	164	C10H12O2
13160330	D20	51351	284	C17H16O4
13160330	D21	72715	605	C23H28N2O5.C6H8O7
13160330	D22	85235	246	C15H18O3

13160330	E03	2952	300	C20H28O2
13160330	E04	13252	515	C22H23ClN2O8.ClH
13160330	E05	31867	398	C23H26O6
13160330	E06	58368	640	C26H34O7.C12H23N
13160330	E07	89671	280	C16H24O4
13160330	E08	145118	460	C25H33NO7
13160330	E09	250429	364	C20H28O6
13160330	E10	305222	494	C30H39NO5
13160330	E11	350085	270	C16H14O4
13160330	E12	661755	877	C46H48N2O8.2C2H4O2
13160330	E13	4143	433	C9H9I2NO3
13160330	E14	7535	985	C49H76O20
13160330	E15	10105	355	C21H25NO4
13160330	E16	16631	154	C7H6O4
13160330	E17	23615	228	C9H12N2O5
13160330	E18	32743	339	C17H25NO6
13160330	E19	36437	376	C22H32O5
13160330	E20	46728	376	C22H32O5
13160330	E21	63946	858	C45H79NO14
13160330	E22	79404	380	C20H28O7
13160330	F03	7668	304	C16H32O5
13160330	F04	26258	394	C23H22O6
13160330	F05	36398	304	C15H12O7
13160330	F06	72116	354	C21H26N2O3
13160330	F07	122023	1111	C54H90N6O18
13160330	F08	177406	591	C34H54O8
13160330	F09	284437	248	C15H20O3
13160330	F10	332876	318	C20H30O3
13160330	F11	400978	248	C15H20O3
13160330	F12	2347	176	C6H8O6
13160330	F13	6435	302	C20H30O2
13160330	F14	8751	86	C4H6O2
13160330	F15	14135	248	C14H16O4
13160330	F16	19990	770	C40H51NO14
13160330	F17	26327	242	C15H14O3
13160330	F18	34758	313	C17H15NO5
13160330	F19	43339	232	C15H20O2
13160330	F20	56410	348	C16H20N4O5
13160330	F21	72861	305	C17H23NO4
13160330	F22	85998	265	C8H15N3O7
13160330	G03	3053	1255	C62H86N12O16
13160330	G04	14975	497	C25H36O10
13160330	G05	32192	367	C20H17NO6

13160330	G06	60387	393	C24H27NO4
13160330	G07	94600	348	C20H16N2O4
13160330	G08	150817	942	C47H75NO18
13160330	G09	250430	421	C26H28O5
13160330	G10	307981	434	C26H26O6
13160330	G11	361902	264	C15H20O4
13160330	G12	719655	379	C22H25N3O3
13160330	G13	4586	356	C20H20O6
13160330	G14	7606	158	C4H6N4O3
13160330	G15	11866	238	C16H14O2
13160330	G16	18334	828	C42H53NO16
13160330	G17	23878	276	C15H16O5
13160330	G18	32944	540	C28H38N2O4.2ClH
13160330	G19	36508	471	C26H30O8
13160330	G20	47147	323	C20H25N3O
13160330	G21	67392	152	C10H16O
13160330	G22	81463	400	C22H28N2O5
13160330	H03	8519	260	C14H12O5
13160330	H04	26271	261	C7H15Cl2N2O2P
13160330	H05	42038	261	C15H19NO3
13160330	H06	76022	429	C20H19NO6.C2H4O2
13160330	H07	122224	639	C33H58N4O8
13160330	H08	177858	824	C43H49N7O10
13160330	H09	285116	1649	C71H81N19O18S5
13160330	H10	333856	1337	C67H96N2O24.Na
13160330	H11	401005	354	C21H22O5
13160330	H12	2802	186	C9H14O4
13160330	H13	6832	150	C10H14O
13160330	H14	8797	393	C24H40O4
13160330	H15	14664	152	C5H4N4O2
13160330	H16	20103	170	C7H6O5
13160330	H17	27425	175	C6H13N3O3
13160330	H18	35550	431	C20H23NO4.C2H2O4
13160330	H19	43871	141	C8H15NO
13160330	H20	59258	192	C7H12O6
13160330	H21	72862	364	C22H24N2O3
13160330	H22	86005	586	C29H31NO12
13160330	I03	5113	420	C25H24O6
13160330	I04	15780	457	C20H27NO11
13160330	I05	32979	341	C20H23NO4
13160330	I06	62709	546	C28H27N5O7
13160330	I07	96911	242	C12H10N4O2
13160330	I08	153858	692	C34H46ClN3O10

13160330	I09	255109	546	C28H39N3O8
13160330	I10	325014	383	C14H20Cl2N2O6
13160330	I11	369397	282	C15H22O5
13160330	I12	824	200	C10H16O4
13160330	I13	5036	194	C8H10N4O2
13160330	I14	7616	174	C6H6O6
13160330	I15	11905	242	C15H14O3
13160330	I16	18805	164	C10H12O2
13160330	I17	24819	414	C22H22O8
13160330	I18	32984	369	C21H23NO5
13160330	I19	36693	332	C20H28O4
13160330	I20	50131	464	C23H28O10
13160330	I21	69187	276	C18H16N2O
13160330	I22	83433	328	C19H20O5
13160330	J03	9665	282	C16H26O4
13160330	J04	26326	242	C15H14O3
13160330	J05	45383	506	C25H22N4O8
13160330	J06	82151	564	C27H29NO10.ClH
13160330	J07	122750	561	C29H40N2O9
13160330	J08	180515	196	C10H12O4
13160330	J09	287088	511	C28H30O9
13160330	J10	337783	362	C21H30O5
13160330	J11	407286	302	C14H6O8
13160330	J12	2835	321	C18H34O3.Na
13160330	J13	7521	531	C30H42O8
13160330	J14	8973	170	C10H18O2
13160330	J15	14665	136	C5H4N4O
13160330	J16	20264	347	C10H14N5O7P
13160330	J17	28841	196	C4H3N3O4.K
13160330	J18	35676	220	C11H8O5
13160330	J19	44138	260	C6H13O9P
13160330	J20	59263	322	C14H10O9
13160330	J21	72917	396	C23H28N2O4
13160330	J22	88466	306	C14H10O8
13160330	K03	5159	641	C32H32O14
13160330	K04	22070	306	C17H22O5
13160330	K05	32982	368	C21H20O6
13160330	K06	63701	291	C12H13N5O4
13160330	K07	105388	671	C35H58O12
13160330	K08	157035	248	C15H20O3
13160330	K09	263164	314	C14H23N5O.ClH
13160330	K10	325319	1112	C57H89N7O15
13160330	K11	375294	234	C15H22O2

13160330	K12	1115	192	C7H12O6
13160330	K13	5379	271	C18H38O
13160330	K14	7652	267	C10H13N5O4
13160330	K15	12444	182	C9H10O4
13160330	K16	19028	222	C15H10O2
13160330	K17	24872	258	C15H14O4
13160330	K18	33410	462	C27H27NO6
13160330	K19	38010	309	C20H36O2
13160330	K20	50132	446	C23H26O9
13160330	K21	70931	451	C29H38O4
13160330	K22	83436	344	C20H24O5
13160330	L03	11440	390	C20H19NO5.ClH
13160330	L04	29854	637	C35H44N2O9
13160330	L05	45923	216	C12H8O4
13160330	L06	85236	262	C15H18O4
13160330	L07	122819	657	C32H32O13S
13160330	L08	209870	547	C28H50O10
13160330	L09	292567	748	C40H68O11.Na
13160330	L10	339555	905	C47H68O17
13160330	L11	407306	354	C21H26N2O3
13160330	L12	3071	152	C8H8O3
13160330	L13	7525	693	C36H52O13
13160330	L14	9170	373	C20H23NO4S
13160330	L15	14974	396	C20H28O8
13160330	L16	21725	244	C15H20N2O
13160330	L17	30238	148	C4H4O6
13160330	L18	36294	248	C14H16O4
13160330	L19	44175	292	C18H12O4
13160330	L20	59729	361	C13H19N3O5S2
13160330	L21	72942	295	C8H15N5O.H2O4S
13160330	L22	89937	299	C15H25NO5
13160330	M03	5366	413	C22H23NO7
13160330	M04	22842	268	C15H8O5
13160330	M05	35611	905	C45H73NO15.ClH
13160330	M06	67574	923	C46H56N4O10.H2O4S
13160330	M07	114344	276	C15H16O5
13160330	M08	169627	768	C40H49NO14
13160330	M09	270914	447	C24H30O8
13160330	M10	330753	587	C31H38O11
13160330	M11	376248	475	C31H22O5
13160330	M12	2080	504	C18H32O16
13160330	M13	5863	146	C8H6N2O
13160330	M14	8625	174	C10H6O3

13160330	M15	12865	405	C20H24N2O2.BrH
13160330	M16	19038	344	C19H20O6
13160330	M17	24951	350	C21H22N2O3
13160330	M18	34202	503	C30H46O6
13160330	M19	38270	1197	C58H84O26
13160330	M20	50393	224	C15H12O2
13160330	M21	72138	579	C32H38N2O8
13160330	M22	83439	334	C20H30O4
13160330	N03	11926	341	C17H11NO7
13160330	N04	30552	410	C24H26O6
13160330	N05	51001	842	C42H67NO16
13160330	N06	85239	262	C15H18O4
13160330	N07	129536	310	C15H18O7
13160330	N08	210236	334	C20H30O4
13160330	N09	301683	262	C13H10O6
13160330	N10	345647	547	C30H26O10
13160330	N11	614552	189	C8H15NO4
13160330	N12	3590	514	C20H23N7O7.Ca
13160330	N13	7532	969	C49H76O19
13160330	N14	9248	194	C6H10O7
13160330	N15	15307	322	C20H22N2O2
13160330	N16	21728	335	C19H16N2.HNO3
13160330	N17	30625	411	C21H33NO7
13160330	N18	36354	507	C20H23NO5.C4H6O6
13160330	N19	45384	521	C26H24N4O8
13160330	N20	61809	398	C18H24NO4.Br
13160330	N21	75527	358	C20H22O6
13160330	N22	90636	907	C46H56N4O9.H2O4S
13160331	C03	93047	229	C9H11NO6
13160331	C04	100290	606	C30H35NO10.ClH
13160331	C05	111041	189	C9H7N3O2
13160331	C06	123977	551	C30H46O9
13160331	C07	143648	346	C12H15N5O5.ClH
13160331	C08	172924	521	C26H32O11
13160331	C09	237671	1330	C63H87N13O19
13160331	C10	266535	307	C14H13NO7
13160331	C11	291312	519	C27H34O10
13160331	C12	330917	237	C9H11N5O3
13160331	C13	403148	398	C22H22O7
13160331	C14	526417	1101	C51H64N12O12S2
13160331	C15	785154	334	C21H22N2O2
13160331	C16	785166	268	C10H12N4O5
13160331	C17	785178	425	C24H28N2O5

13160331	D03	99791	221	C5H12N2O3.2ClH
13160331	D04	106486	786	C40H51NO15
13160331	D05	121849	416	C22H25NO5S
13160331	D06	136035	673	C34H48N4O10
13160331	D07	156219	563	C29H38O11
13160331	D08	186301	621	C31H41ClN2O9
13160331	D09	255112	509	C28H28O9
13160331	D10	269756	623	C33H38N4O6.ClH
13160331	D11	314018	420	C23H33NO6
13160331	D12	354844	379	C14H25N3O9
13160331	D13	616348	182	C10H14O3
13160331	D14	785148	240	C12H20N2O3
13160331	D15	785160	260	C15H16O4
13160331	D16	785172	385	C21H23NO6
13160331	D17	785184	328	C17H28O6
13160331	E03	93373	292	C17H24O4
13160331	E04	100858	635	C32H43ClN2O9
13160331	E05	112906	433	C19H26N2.C4H6O6
13160331	E06	127445	321	C20H19NO3
13160331	E07	145150	350	C21H22N2O3
13160331	E08	172946	360	C18H16O8
13160331	E09	244387	272	C11H13NO2.BrH
13160331	E10	267033	701	C36H48N2O12
13160331	E11	292222	246	C15H18O3
13160331	E12	332294	812	C42H53NO15
13160331	E13	403169	773	C40H48N6O10
13160331	E14	785143	563	C29H38O11
13160331	E15	785155	263	C11H13N5O3
13160331	E16	785167	429	C23H27NO7
13160331	E17	785179	406	C22H30O7
13160331	F03	99792	595	C36H38N2O6
13160331	F04	106969	389	C20H18N2O5.Na
13160331	F05	121859	248	C9H16N2O6
13160331	F06	136044	242	C10H14N2O3S
13160331	F07	156236	755	C40H54N2O12
13160331	F08	208734	823	C43H54N2O14
13160331	F09	259968	428	C25H33N3O.ClH
13160331	F10	269760	1085	C52H76O24
13160331	F11	316458	561	C29H36O11
13160331	F12	355637	439	C21H20F3NO6
13160331	F13	642099	448	C22H24O10
13160331	F14	785149	410	C23H26N2O5
13160331	F15	785161	314	C15H10N2O6

13160331	F16	785173	352	C21H24N2O3
13160331	F17	785185	236	C12H12O5
13160331	G03	93674	490	C28H43NO6
13160331	G04	100880	846	C42H55NO17
13160331	G05	112907	1572	C61H88N18O21S2.H2O4S
13160331	G06	127473	565	C29H40O11
13160331	G07	145612	570	C27H21Cl2N3O7
13160331	G08	177383	495	C25H34O10
13160331	G09	247562	781	C41H64O14
13160331	G10	269146	244	C8H12N4O5
13160331	G11	292463	341	C16H23NO7
13160331	G12	335989	315	C18H21NO4
13160331	G13	407308	379	C22H21NO5
13160331	G14	785144	487	C28H38O7
13160331	G15	785156	715	C38H34O14
13160331	G16	785168	542	C28H31NO10
13160331	G17	785180	487	C20H26N2O8S2
13160331	H03	99794	696	C40H45N3O6S
13160331	H04	106995	375	C23H34O4
13160331	H05	121860	364	C16H13NO4.BrH
13160331	H07	159632	236.22	C12H12O5
13160331	H08	216128	412	C23H28N2O5
13160331	H09	265211	867	C47H78O14
13160331	H10	276382	549	C28H36O11
13160331	H11	327993	268	C11H16N4O4
13160331	H12	359079	730	C37H47NO14
13160331	H13	132791	256	C8H17N3O4.ClH
13160331	H14	785150	535	C27H34O11
13160331	H15	785162	401	C21H23NO5S
13160331	H16	785174	354	C19H14O7
13160331	H17	785186	456	C29H45NO3
13160331	I03	95099	418	C22H26O8
13160331	I04	102816	483	C28H34O7
13160331	I05	113087	349	C18H23NO6
13160331	I06	128487	302	C14H19NO4.ClH
13160331	I07	146396	302	C12H18N2O7
13160331	I08	179834	307	C17H25NO4
13160331	I09	248605	549	C29H40O10
13160331	I10	269148	350	C19H26O6
13160331	I11	294408	478	C29H35NO5
13160331	I12	337851	523	C18H22N2O4.C6H8O7
13160331	I13	407806	510	C27H43NO8
13160331	I14	785145	333	C16H19N3O3S

13160331	I15	785157	298	C17H14O5
13160331	I16	785169	296	C16H24O5
13160331	I17	785181	316	C16H12O7
13160331	J03	99799	697	C35H37ClN2O11
13160331	J04	107041	156	C7H8O4
13160331	J05	121865	391	C23H34O5
13160331	J06	138320	805	C40H52O17
13160331	J07	165563	248	C15H20O3
13160331	J08	218321	580	C27H29NO11.ClH
13160331	J09	265450	381	C22H23NO5
13160331	J10	278619	325	C12H15N5O4S
13160331	J11	328166	229	C12H11N3O2
13160331	J12	361792	482	C25H43N3O6
13160331	J13	178249	433	C25H36O6
13160331	J14	785151	486	C25H26O10
13160331	J15	785163	364	C20H28O6
13160331	J16	785175	549	C29H40O10
13160331	J17	785187	374	C22H30O5
13160331	K03	96021	291	C14H13NO6
13160331	K04	104943	357	C19H23N3O4
13160331	K05	113497	291	C12H13N5O4
13160331	K06	129230	354	C21H26N2O3
13160331	K07	147340	325	C19H19NO4
13160331	K08	180516	326	C19H18O5
13160331	K09	248958	1665	C72H85N19O18S5
13160331	K10	269753	594	C30H51N5O7
13160331	K11	295426	410	C21H30O8
13160331	K12	343256	519	C27H34O10
13160331	K13	601422	561	C30H44N2O8
13160331	K14	785146	785	C42H68N6O6S
13160331	K15	785158	469	C22H22F3NO5S
13160331	K16	785170	293	C19H19NO2
13160331	K17	785182	297	C18H19NO3
13160331	L03	99804	325	C19H19NO4
13160331	L04	107453	311	C18H17NO4
13160331	L05	123383	311	C18H17NO4
13160331	L06	141538	313	C19H23NO3
13160331	L07	169517	596	C36H40N2O6
13160331	L08	227186	337	C19H15NO5
13160331	L09	266032	367	C21H21NO5
13160331	L10	281245	297	C18H19NO3
13160331	L11	328426	355	C20H21NO5
13160331	L12	365793	321	C19H15NO4

13160331	L13	256942	339	C20H21NO4
13160331	L14	785152	608	C37H40N2O6
13160331	L15	785164	313	C19H23NO3
13160331	L16	785176	351	C20H17NO5
13160331	L17	785188	263	C17H13NO2
13160331	M03	98542	279	C18H17NO2
13160331	M04	105827	338	C21H26N2O2
13160331	M05	114341	351	C20H17NO5
13160331	M06	135962	339	C20H21NO4
13160331	M07	148790	622	C38H42N2O6
13160331	M08	184398	297	C18H19NO3
13160331	M09	250682	295	C19H21NO2
13160331	M10	269754	309	C18H15NO4
13160331	M11	302979	325	C20H23NO3
13160331	M12	349155	325	C19H19NO4
13160331	M13	607097	337	C19H15NO5
13160331	M14	785147	385	C21H23NO6
13160331	M15	785159	329	C19H23NO4
13160331	M16	785171	341	C20H23NO4
13160331	M17	785183	355	C21H25NO4
13160331	N03	99843	327	C19H21NO4
13160331	N04	108088	281	C18H19NO2
13160331	N05	123389	325	C19H19NO4
13160331	N06	142227	281	C18H19NO2
13160331	N07	170365	279	C18H17NO2
13160331	N08	236580	476	C32H32N2O2
13160331	N09	266071	325	C19H19NO4
13160331	N10	283445	325	C19H19NO4
13160331	N11	330500	281	C18H19NO2
13160331	N12	376128	413	C26H23NO4
13160331	N13	320301	610	C37H42N2O6
13160331	N14	785153	295	C19H21NO2
13160331	N15	785165	476	C32H32N2O2
13160331	N16	785177	281	C18H19NO2
13160331	N17	785189	341	C20H23NO4

S2 Table. Percent inhibition of larval motility for the different timepoints tested for compound NSC133100, against the susceptible isolate of *H. contortus.* Data for 3 technical replicates were averaged for analysis.

Concentration (μM)	Initial	1 hour	4 hours	24 hours	48 hours	72 hours	96 hours
125	-8.9%	4.3%	-12.0%	3.2%	23.2%	3.1%	19.2%
31.25	-64.8%	10.6%	7.1%	9.0%	35.2%	19.9%	28.7%
7.81	-74.0%	27.5%	9.1%	25.3%	35.3%	32.1%	38.0%
1.95	-159.9%	-57.6%	-16.4%	-8.4%	-37.5%	-49.0%	-50.7%
0.49	-118.5%	-29.1%	4.3%	8.0%	-9.5%	-14.9%	-9.0%
0.12	-93.9%	-7.4%	14.9%	22.9%	8.7%	26.1%	7.4%

S3 Table. Percent inhibition of larval motility for the different timepoints tested for Compound NSC177383, against the susceptible isolate of *H. contortus.* Data for 3 technical replicates were averaged for analysis.

Concentration (μM)	Initial	1 hour	4 hours	24 hours	48 hours	72 hours	96 hours
125	22.02%	42.55%	10.81%	16.94%	42.97%	27.79%	41.45%
31.25	28.34%	14.47%	38.95%	18.83%	26.89%	16.58%	-23.43%
7.81	22.03%	26.94%	26.72%	22.63%	34.44%	14.00%	-53.76%
1.95	21.82%	30.56%	33.37%	18.80%	14.16%	-3.70%	-23.78%
0.49	-11.69%	25.94%	18.27%	23.09%	12.98%	-5.94%	-72.91%
0.12	-1.62%	29.41%	4.95%	28.20%	2.41%	4.89%	-16.32%

S4 Table. Percent inhibition of larval motility for the different timepoints tested for Compound NSC133100, against the MDR isolate of *H. contortus*. Data for 3 technical replicates were averaged for analysis.

**Concentration (µM)**	**Initial**	**1 hour**	**4 hours**	**24 hours**	**48 hours**	**72 hours**	**96 hours**
500	5.8%	33.9%	-2.0%	-0.5%	22.2%	12.1%	18.5%
250	-11.5%	38.9%	-5.6%	-12.4%	28.6%	-1.2%	0.9%
125	-12.3%	33.8%	3.0%	-6.2%	18.4%	14.0%	26.6%
62.5	-25.7%	13.5%	-11.1%	-12.6%	-2.3%	-9.5%	-1.6%
31.25	-21.7%	6.3%	1.6%	0.6%	11.9%	3.1%	5.5%
15.625	-7.4%	6.1%	6.9%	-6.8%	-11.1%	-11.7%	-3.4%

S5 Table. Percent inhibition of larval motility for the different timepoints tested for Compound NSC177383, against the MDR isolate of *H. contortus*. Data for 3 technical replicates were averaged for analysis.

**Concentration (µM)**	initial	1 hour	4 hours	24 hours	48 hours	72 hours	96 hours
**500**	36.4%	27.1%	-12.9%	26.6%	49.1%	32.3%	21.4%
**250**	43.9%	28.8%	-42.2%	30.2%	44.8%	46.1%	31.2%
**125**	22.5%	26.8%	-34.1%	20.5%	31.1%	4.1%	5.7%
**62.5**	16.5%	3.9%	-28.6%	23.5%	26.0%	19.1%	14.7%
**31.25**	23.2%	2.3%	5.2%	13.8%	41.2%	25.5%	17.1%
**15.625**	19.9%	-22.8%	-24.6%	15.6%	3.0%	-5.8%	-22.4%

S6 Table. Percent inhibition of larval motility for the different timepoints tested for Compound NSC145612, against the MDR isolate of *H. contortus.* Data for 3 technical replicates were averaged for analysis.

**Concentration (µM)**	initial	1 hour	4 hours	24 hours	48 hours	72 hours	96 hours
500	9.3%	-19.2%	0.4%	-12.0%	14.1%	9.6%	-5.1%
250	25.9%	13.0%	47.0%	-8.6%	13.8%	9.2%	12.8%
125	21.6%	31.7%	34.3%	15.7%	8.1%	3.0%	-6.9%
62.5	10.1%	16.7%	32.0%	10.4%	14.1%	18.3%	3.8%
31.25	21.0%	20.1%	-11.2%	27.2%	21.9%	43.2%	3.9%
15.625	20.3%	-10.3%	7.3%	7.0%	8.1%	23.0%	17.4%

S7 Table. PCR Primers for amplification of PMTs coding fragments for cloning in pET15b expression vector

Gene	Forward primer	Reverse Primer
AcPMT1	5′-*CTCGAG*ATGGAGAGGAGTGTGGA-3′	5′-*GGATCC***TTA**CAACTCTATGAATAAGA-3′
AcPMT2	5′-*CTCGAG*ATGCCAGCCGCTGAGCAGCA-3′	5′-*CTCGAG***TTA**CTGTGGTTTCGAAGCCA-3′
AsPMT	5′-*CTCGAG*ATGACCGAAGCAATTCGAC-3′	5′-*GGATCC***TCA**CATATGCGCTCTTATTA-3′
DvPMT1	5′-*CTCGAG*ATGAGCCAAGGAATGAGACA-3′	5′-*GGATCC***TTA**GAGTGTGGCACTGATGA-3′
DvPMT2	5′-*CTCGAG*ATGCGGACCGGTCAAAGAAT-3′	5′-*GGATCC***CTA**CTGAGGTTTACTGGCCA-3′
OdPMT	5′-*CTCGAG*ATGATTTTTCAAAAACGCTTCACTAC-3′	5′-*GGATCC***TTA**GAGTGAAGCGTTGATGA-3′
TcPMT	5′-*CATATG*ATGCCAAGATTGAATCAGAA-3′	5′-*GGATCC***TTA**AAGTGGTTTGGTTGCAA-3′

Italicized: Restriction site (XhoI/BamHI); Underlined: Start codon; **Bold**: stop codon

S8 Table. PCR Primers for amplification of PMTs coding fragments for cloning in pYES2.1 expression vector

Gene	Forward primer	Reverse Primer
AcPMT1	5′-*GCC<u>ATG</u>G*AGAGGAGTGTGGA-3′	5′-**TTA**CAACTCTATGAATAAGA-3′
AcPMT2	5′-*GCC<u>ATG</u>G*CAGCCGCTGAGCAGCA-3′	5′-**TTA**CTGTGGTTTCGAAGCCA-3′
AsPMT	5′-*GCC<u>ATG</u>G*CCGAAGCAATTCGAC-3′	5′-**TCA**CATATGCGCTCTTATTA-3′
DvPMT1	5′-*GCC<u>ATG</u>G*GCCAAGGAATGAGACA-3′	5′-*GGATCC***TTA**GAGTGTGGCACTGATGA-3′
DvPMT2	5′-*GCC<u>ATG</u>G*GGACCGGTCAAAGAAT-3′	5′-**CTA**CTGAGGTTTACTGGCCA-3′
OdPMT	5′-*GCC<u>ATG</u>G*TTTTTCAAAAACGCTTCACTAC-3′	5′-**TTA**GAGTGAAGCGTTGATGA-3
TcPMT	5′-*GCC<u>ATG</u>G*CAAGATTGAATCAGAA-3′	5′-**TTA**AAGTGGTTTGGTTGCAA-3
HcPMT1	5′-*GCC<u>ATG</u>G*CGGCTGAGGTGCGACGGGATT-3′	5′-**TTA**AAGTGAAGCCTTGATCA-3′
HcPMT2	5′-*GCC<u>ATG</u>G*CTGCCGTTGAACGACAACTGATT-3′	5′-**TTA**TTGTGGCTTGACAGCAGCGAA-3′

Italicized: Restriction site Kozak sequence; <u>Underlined</u>: Start codon; **Bold**: stop codon
